# Rewilding the world's large carnivores

**DOI:** 10.1098/rsos.172235

**Published:** 2018-03-14

**Authors:** Christopher Wolf, William J. Ripple

**Affiliations:** Global Trophic Cascades Program, Department of Forest Ecosystems and Society, Oregon State University, Corvallis, OR 97331, USA

**Keywords:** predator, geographical range, reintroduction, intact guild, carnivore guild, ecological effectiveness

## Abstract

Earth's terrestrial large carnivores form a highly endangered group of species with unique conservation challenges. The majority of these species have experienced major geographical range contractions, which puts many of them at high risk of extinction or of becoming ecologically ineffective. As a result of these range contractions and the associated loss of intact predator guilds, the ecological effects of these species are now far less widespread and common, with inevitable consequences for ecosystem function. Rewilding—which includes reintroducing species into portions of their former ranges—is an important carnivore conservation tool and means for restoring top-down ecological regulation. We conducted a global analysis of potential reintroduction areas. We first considered protected areas where one or more large carnivore species have been extirpated, identifying a total of 130 protected areas that may be most suitable for carnivore reintroduction. These protected areas include sites in every major world region, and are most commonly found in Mongolia (*n* = 13), Canada (*n* = 11), Thailand (*n* = 9), Namibia (*n* = 6), Indonesia (*n* = 6) and Australia (*n* = 6). We considered the sizes of protected areas, their levels of protection, the extent of human impacts within and around the protected areas, and the status of prey species in the protected areas. Finally, we used the ‘last of the wild’ approach to identify contiguous low human footprint regions within the former ranges of each species, identifying an additional 150 areas which could be the focus of conservation efforts to create conditions conducive to reintroductions. These low footprint regions were most commonly found in the USA (*n* = 14), Russia (*n* = 14), Canada (*n* = 10), China (*n* = 9) and Mauritania (*n* = 8). Together, our results show the global-scale potential for carnivore rewilding projects to both conserve these species and provide critical ecological and social benefits.

## 1. Introduction

**Figure 1.**
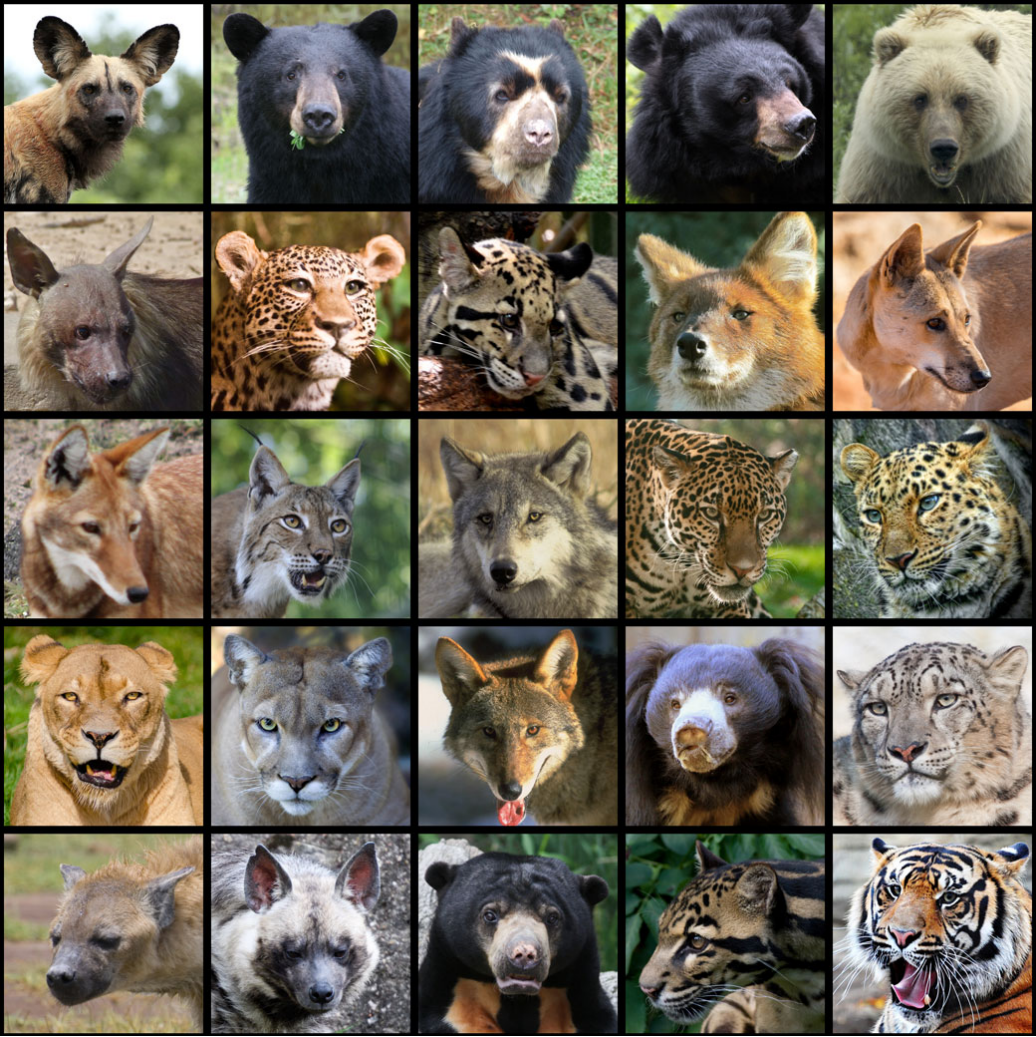
The 25 terrestrial large carnivore species in our analysis (table 1). From left to right, the species are: first row—African wild dog, American black bear, Andean black bear, Asiatic black bear, brown bear; second row—brown hyaena, cheetah, clouded leopard, dhole, dingo; third row—Ethiopian wolf, Eurasian lynx, gray wolf, jaguar, leopard; fourth row—lion, puma, red wolf, sloth bear, snow leopard; fifth row—spotted hyaena, striped hyaena, sun bear, Sunda clouded leopard, tiger. Scientific names are given in table 1 and photo credits are given in electronic supplementary material, table S1.

Earth's terrestrial large carnivores are a charismatic, highly endangered group of species. In total, 64% of these species are threatened with extinction and 80% have declining population trends (table 1, figure 1). Major threats to large carnivore survival include habitat loss and fragmentation, persecution by humans (often due to livestock-related conflict), utilization of body parts for traditional medicine or trophies, and loss of prey base [7,8]. These threats have together diminished the ranges of many species, often to the point where they are highly endangered. Sixty per cent of these species have lost more than half of their historic ranges in the last 500 years [9]. As a result of these range contractions and associated population declines, the important ecological effects of large carnivores have been lost from much of the world [10]. For example, previous studies have documented the potential for large carnivores to trigger trophic cascades by limiting herbivore or mesopredator densities, indirectly benefitting a wide variety of plants and animals [7,11].

**Table 1.** The 25 large carnivore species in our analysis. From left to right, the variables shown are taxonomic family, species' scientific name, species’ common name, IUCN Red List category (LC, least concern; NT, near threatened; VU, vulnerable; EN, endangered; CR, critically endangered), IUCN Red List species' population trend, percentage of species’ range lost and whether a reintroduction of the species has been documented (with source if applicable).

family	scientific name	common name	category	trend	range lost (%)	reintroduced?
Canidae	*Canis rufus*	red wolf	CR	increasing	>99	yes [1]
Canidae	*Canis simensis*	Ethiopian wolf	EN	decreasing	99	no
Felidae	*Panthera tigris*	tiger	EN	decreasing	95	no
Felidae	*Panthera leo*	lion	VU	decreasing	94	yes [2]
Canidae	*Lycaon pictus*	African wild dog	EN	decreasing	93	yes [2]
Felidae	*Acinonyx jubatus*	cheetah	VU	decreasing	92	yes [2]
Canidae	*Cuon alpinus*	dhole	EN	decreasing	82	no
Felidae	*Panthera pardus*	leopard	VU	decreasing	79	yes [2]
Felidae	*Panthera uncia*	snow leopard	EN	decreasing	78	yes [3]
Ursidae	*Tremarctos ornatus*	Andean black bear	VU	decreasing	75	yes [4]
Ursidae	*Ursus thibetanus*	Asiatic black bear	VU	decreasing	64	yes [5]
Felidae	*Neofelis nebulosa*	clouded leopard	VU	decreasing	64	no
Felidae	*Neofelis diardi*	Sunda clouded leopard	VU	decreasing	51	no
Felidae	*Panthera onca*	jaguar	noT	decreasing	50	no
Ursidae	*Helarctos malayanus*	sun bear	VU	decreasing	50	no
Ursidae	*Ursus arctos*	brown bear	LC	stable	42	yes [1]
Ursidae	*Ursus americanus*	American black bear	LC	increasing	39	yes [3]
Ursidae	*Melursus ursinus*	sloth bear	VU	decreasing	39	no
Felidae	*Puma concolor*	puma	LC	decreasing	32	yes [1]
Hyaenidae	*Hyaena brunnea*	brown hyaena	noT	decreasing	27	yes [2]
Canidae	*Canis lupus*	gray wolf	LC	stable	26	yes [6]
Hyaenidae	*Crocuta crocuta*	spotted hyaena	LC	decreasing	24	yes [2]
Hyaenidae	*Hyaena hyaena*	striped hyaena	noT	decreasing	15	no
Felidae	*Lynx lynx*	Eurasian lynx	LC	stable	12	yes [3]
Canidae	*Canis dingo*	dingo	VU	decreasing	12	no

While the loss of large carnivores from major portions of their historic ranges has altered the functioning of nearly all of Earth's ecosystems, recent trends have not been entirely negative. The natural range expansions of the gray wolf, brown bear and Eurasian lynx in parts of Europe and of the gray wolf in the USA suggest that it is possible for large carnivores and humans to coexist when human tolerance and policy are favourable (i.e. when poverty is rare, people are educated, and reliance on subsistence agriculture and hunting is limited) [12–14]. Unfortunately, for some species like the endangered Ethiopian wolf (greater than 99% of historical range lost), relying on natural range expansions and conserving them where they currently reside may not be sufficient to ensure their survival. However, there is another option: rewilding—planned reintroductions of large carnivores back into parts of their historic ranges from which they have been lost [1]. Benefits of large carnivore reintroductions include reducing species extinction risk [15], providing opportunities for natural range expansions beyond the reintroduction area (e.g. the expansion of gray wolves into Washington, Oregon and California following their reintroduction into Idaho), providing opportunities for wildlife viewing tourism [16], and potentially restoring ecosystem function by re-establishing predators' ecological effects [17]. Such benefits must be weighed carefully against potential harm to animals being translocated, possibly lower human tolerance for reintroduced rather than naturally returning carnivores, and the risks that large carnivores can pose to humans, including potential loss of livestock or pets, direct attacks and disease transmission [14].

In recent decades, there has been some research on the topic of carnivore reintroductions, which we summarize to provide context for our analysis. Most of this work relates to the question of what makes a carnivore reintroduction attempt successful. For example, protected area size has been found to be closely linked to the likelihood of large carnivore persistence, with one study reporting critical reserve sizes necessary for 50% probability of persistence ranging from 36 km^2^ for the American black bear to 3606 km^2^ for the African wild dog [18]. Carnivores in smaller protected areas may be more vulnerable to negative edge effects just outside reserve borders involving hunting or trapping by humans and conflict with humans over livestock and agriculture, particularly when efforts like fence construction are not taken to reduce conflict in surrounding buffer zones [18,19]. For example, in the Western USA, extensive grazing of livestock on public lands reduces the amount of food available for wild ungulates, leading to a reduction in prey base for carnivores [20]. Another important consideration for carnivore reintroductions is the availability of suitable prey, without which large carnivores cannot persist [8,21]. Food web-based modelling of reintroductions highlights the role of priority effects where established competitors can cause reintroductions to fail unless the size of the reintroduced population is sufficiently large [21]. The life-history traits of large carnivores—relatively slow growth rates, a tendency to travel long distances, possibility for conflict with humans—can make reintroducing these species exceptionally challenging [21]. In a study of translocation success rates, carnivore and herbivore translocations had success rates of 48% (*n* = 40) and 77% (*n* = 145), respectively [22].

Motivated by the urgent need to address carnivore decline, we conducted the first comprehensive, global analysis of large carnivore reintroduction possibilities. Our primary goal was, for each large carnivore species, to identify specific protected areas and other areas where the likelihood of successful reintroduction may be high, providing data on likely predictors of reintroduction success for each region. To accomplish this broad goal, we defined the following objectives for our analysis (at the level of candidate reintroduction areas). Our first set of objectives was to determine geographical area, geographical region (e.g. continent), level of protection (protected areas only), mean human footprint (a spatial measure of human impacts on the environment) and availability of suitable prey for each large carnivore species—all likely predictors of reintroduction success [18,23]. Our second set of objectives was to determine which potential reintroduction efforts would lead to complete large carnivore guilds, which are typically associated with key ecological effects due to emergent impacts on prey, and to manually validate potential reintroduction sites using the literature to confirm extirpation [24]. Taken as a whole, our analysis serves as a preliminary, data-driven assessment of global rewilding possibilities.

## 2. Material and methods

To conduct our rewilding analysis, we considered the 25 large (≥15 kg) terrestrial carnivores (members of the mammalian order Carnivora) for which relatively accurate historic and current range maps were available [9] (table 1). We excluded semi-aquatic large carnivore species like the polar bear (*Ursus maritimus*) because the unique habitat requirements of these species are beyond the scope of our terrestrial-focused analysis. The only exclusively terrestrial large carnivore species that we omitted was the maned wolf (*Chrysocyon brachyurus*), as we could not obtain a suitable historic range map for this species. Using the historic and current range map set from [9], we constructed maps showing the ‘lost range’ of each species—the areas within its historic range that do not overlap its current range. These lost ranges are the foundation of our analysis as they indicate areas where reintroduction may be considered. For all spatial analysis, we used raster maps at 5 km resolution in Behrmann equal area projection. Spatial analysis was carried out in ‘R’ and ArcGIS v.10.1 [25,26].

After identifying the lost ranges of each species, we conducted parallel analyses using two different approaches. For the first approach, we focused on protected areas. We used the World Database on Protected Areas (WDPA)—a global database of protected areas—for this part [27]. We considered only protected areas with polygon-type spatial data available and International Union for Conservation of Nature protected area category Ia (Strict Nature Reserve), Ib (Wilderness Area), II (National Park) or III (National Monument or Feature). These categories correspond to the highest levels of protection and are thus most suitable for large carnivore reintroductions. For each large carnivore species, we determined the set of such protected areas at least partially within its lost range. We excluded protected areas that overlap the species' current range. We used reserve size as the primary criterion to determine the protected areas best suited for reintroduction. Working from largest to smallest, we consulted the literature to see if each reserve was known to contain the large carnivore species. We stopped this process after identifying the six largest reserves for each large carnivore species where its status in the reserve was either absent or unknown. To determine carnivore status within each reserve, we used a Google search consisting of carnivore species common name(s) and the protected area name(s) (exact matches only). To keep this task manageable, we looked only at the first page of search results, visiting any websites that appeared likely to contain the needed information. Admittedly, this approach could result in some inaccuracies, particularly for countries that lack English language literature on their protected areas.

Our second approach to determining areas with high reintroduction potential was based on the 2009 global human footprint map [23]. This map shows the impacts of humans on the environment based on source data including maps of roads, night-time lights, land cover and human population density. Although the exact nature of large carnivore responses to each of these anthropogenic pressures varies, human footprint provides a reasonable starting point to assess where large carnivores are most likely to persist. We used the ‘last of the wild’ methodology to determine large, relatively intact, regions within the lost ranges of each large carnivore species [28]. ‘Last of the wild’ regions are defined as contiguous regions within a bigger region that are in the bottom 10% in terms of human footprint over the bigger region. That is, we first identified the 10% of each lost range with the lowest human footprint. We then determined contiguous regions within this lowest 10% footprint portion of each range. When determining contiguous regions, we defined the neighbourhood of each raster grid cell as the eight (rather than four) closest cells. For our primary analysis involving low footprint regions, we focused on the largest six regions for each carnivore species to match our protected area analysis. To provide context, we also constructed histograms of the human footprint across each large carnivores' historic range split into separate categories for current range and lost range.

After identifying protected areas and low footprint regions for each large carnivore, we conducted parallel analyses using these two datasets to explore where reintroductions may have the greatest likelihood of success. We present results for all categories I–III protected areas and low footprint regions that we identified for each carnivore species, but emphasize results for the six largest protected areas and low footprint regions. For each large carnivore species, in addition to looking at the geographical area and average human footprint of each area (protected area or low footprint region), using the historic range maps, we determined whether or not the large carnivore guild (set of species) becomes complete (relative to approximately AD 1500) if that carnivore species is reintroduced there. We also determined the country and broad-scale geographical region of the site, using the United Nation's M49 standard and treating southeastern Asia and the rest of Asia (denoted ‘Asia’) as separate regions [29]. For low footprint regions that cover multiple countries, we state only the country that contains the largest portion of the region. For low footprint regions only, we report the portion of the region that is protected (i.e. that overlaps categories Ia, Ib, II or III protected areas). Finally, for the hyper carnivore species (large carnivores with diets containing at least 70% meat), when data are available, we report the preferred prey species, threatened preferred prey species, and estimated total number of preferred prey species available at each site [8]. We used the IUCN Red List to obtain range maps of the preferred prey species, considering only regions where each species is classified as ‘Extant’ or ‘Probably Extant.’ We present the results of our analysis using separate scatter plots and tables for each of the two approaches, along with maps showing site locations for all large carnivore species together and separate maps for each large carnivore species.

We used two approaches to assess the connectivity of the protected areas identified for each species. First, for each of these protected areas, we used the 5 km resolution protected area raster map to calculate the distance (accurate to approximately 5 km) to the nearest (other) categories I–III protected area of any size. We summarized results for the six largest protected areas identified for each species. Second, pooling the sets of the six largest protected areas for each species together, we analysed low cost corridors among these protected areas using the ‘Linkage Mapper’ package [30]. Linkage Mapper is designed to construct a map showing the value of each raster grid cell as a corridor for connecting protected (or other ‘core’) areas. This map highlights the corridors that are most important to maintaining connectivity between these large protected areas. In short, this is accomplished by determining adjacent protected areas using least cost paths, building cost-weighted distance rasters for each protected area, and then normalizing and compositing the cost-weighted distance rasters to obtain a single map showing ‘corridor value’ for each grid cell [30]. We treated protected areas separated by more than 500 km as non-adjacent, reflecting the dispersal distances possible for large carnivores [31]. To determine cost-weighted distances, we used human footprint (linearly rescaled to range from 1 to 100) as the ‘resistance’ raster indicating the approximate difficulty for large carnivores to traverse a grid cell. Since cropland is a potential barrier to large carnivore movement (it is associated with lack of native vegetation cover, conflicts over livestock, and diminished wild prey base), we masked out grid cells containing cropland from the resistance map using the Global Cropland Area Database (GCAD) [9,32]. For this analysis, we used the 25 largest protected areas in each species' lost range in order to gain a more complete picture of connectivity.

## 3. Results

**Figure 2.**
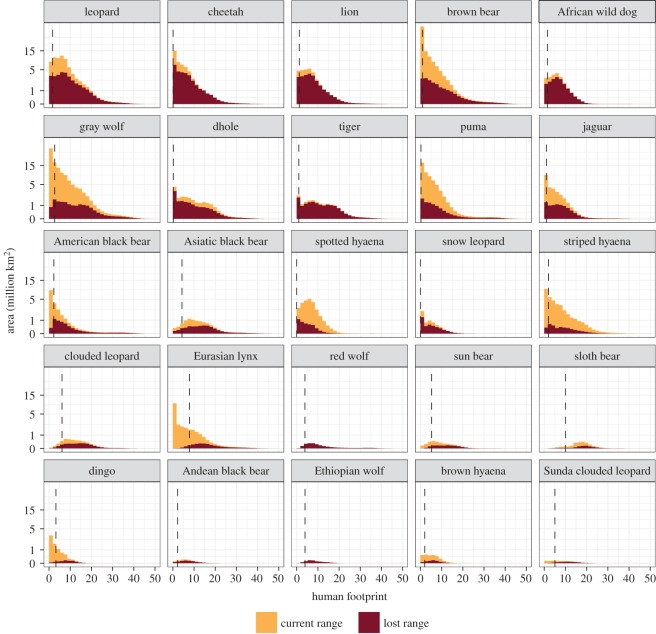
Histograms for human footprint (a spatial measure of human impacts on the environment) across the historic ranges of each large carnivore species. The historic range (both colours together) is split into ‘Current range’ (regions where the carnivore species is still present) and ‘Lost range’ (regions where the carnivore species has been extirpated). The vertical lines indicate thresholds for ‘last of the wild’ regions within lost ranges. That is, the bottom 10% threshold (quantile) for human footprint in the lost range.

We found dramatic variation in human footprint across the lost ranges of the large carnivore species (figure 2). On a scale from 0 to 50, the ‘last of the wild’ (10% quantile thresholds) varied from 0.0 for the snow leopard and spotted hyaena to 10.0 for the sloth bear (figure 2).

**Figure 3.**
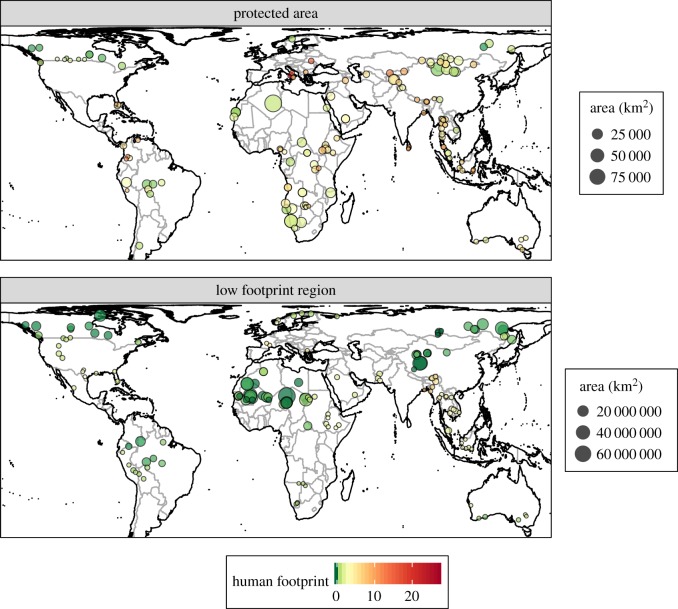
Potential sites for reintroducing large carnivores. (*a*) The locations, areas and mean human footprints of the six largest strictly protected areas within each of the 25 large carnivores' lost ranges (i.e. where the species has been extirpated). (*b*) The same data for the six largest low footprint regions within the lost range of each species. Low footprint regions were determined based on contiguous areas within the ‘last of the wild’ regions of each large carnivore's lost range. ‘Last of the wild’ regions are those in the bottom 10% for human footprint within each species' lost range.

**Figure 4.**
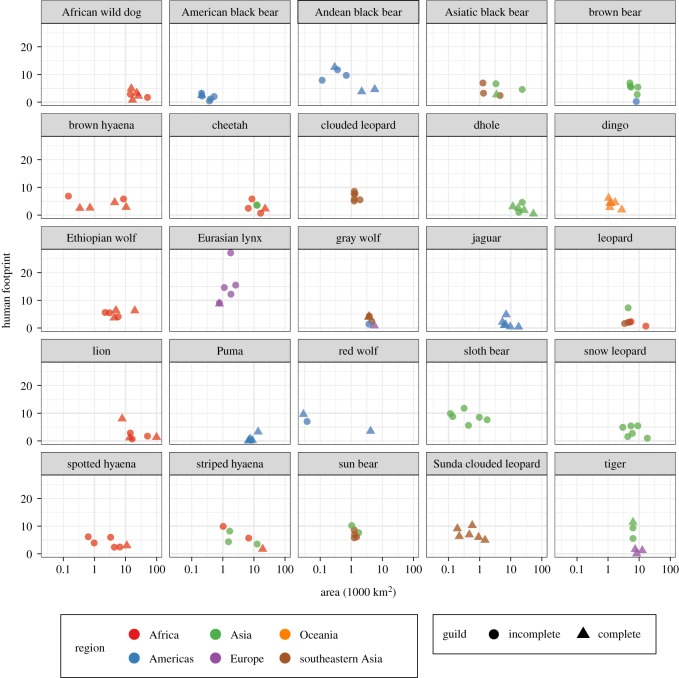
The six largest protected areas inside the ‘lost’ (historic minus current) ranges of each large carnivore species (only three were identified for the red wolf). For each carnivore species, variables shown are mean human footprint across the protected area, region of the world, and whether or not the large carnivore guild becomes complete following reintroduction of the carnivore species. Only strictly protected (IUCN categories I–III) were considered for this analysis. Asia is split into southeastern Asia and the rest of Asia (denoted ‘Asia’).

We identified the six largest protected areas for each large carnivore species (except the red wolf—only three protected areas were identified for this species) where reintroduction may be successful following the implementation of conservation programmes designed to limit ongoing threats to carnivores, possibly including the original causes of extirpation (table 2, figure 3). These form a set of 130 different protected areas globally, with areas ranging from 29 km^2^ for Collier-Seminole (red wolf) to 99 331 km^2^ for Parc Culturel du Tassili (Illizi) (lion) (table 2). They cover all the major regions of the world: Africa (*n* = 34), the Americas (*n* = 28), Asia (excluding southeastern Asia) (*n* = 31), Europe (*n* = 10), Oceania (*n* = 6), and southeastern Asia (*n* = 28) and consist of Strict Nature Reserves (*n* = 20), Wilderness Areas (*n* = 14), National Parks (*n* = 94) and National Monuments or Features (*n* = 2) (figure 3, electronic supplementary material, table S2). These protected areas span 48 countries, and most commonly occur in Mongolia (*n* = 13), Canada (*n* = 11), Thailand (*n* = 9), Namibia (*n* = 6), Indonesia (*n* = 6) and Australia (*n* = 6) (electronic supplementary material, table S2). Fifteen of these protected areas appeared in the top six largest protected areas for two different large carnivore species and one (Dakhla National Park in Morocco) appeared in the top six for three species (cheetah, lion and leopard) (table 2). Of the 147 protected area–large carnivore combinations (six for each species except the red wolf), 59 (40.1%) would result in an intact carnivore guild following reintroduction of the large carnivore there (figure 4, electronic supplementary material, table S2). The average human footprint (across protected areas) was highest for the Eurasian lynx (13.2), sloth bear (12.5) and Asiatic black bear (10.0) and lowest for the puma (3.1), dingo (5.3) and brown hyaena (5.8) (figure 4, electronic supplementary material, table S2). Fifteen of the 25 large carnivore species had known preferred prey species, and the carnivore species with the greatest median (across protected areas) number of preferred prey species available were the jaguar (*n* = 8), gray wolf (*n* = 4), red wolf (*n* = 3), dingo (*n* = 3) and puma (*n* = 2) (electronic supplementary material, table S2).

**Table 2.** For each large carnivore species, the six largest protected areas where it has been extirpated (i.e. where we found no evidence in the literature of its current presence). Carnivores species are listed in alphabetical order by common name. Numbers in parentheses are the range of areas for the six protected areas shown. Protected areas are grouped by country (countries sorted alphabetically). Within countries, protected areas are listed in order of decreasing area. For each species, the largest protected area is shown in bold and the smallest is italicized. Additional protected area data including information on potential inaccuracies is given in electronic supplementary material table S2.

African wild dog (14 273–50 985 km^2^): Angola (National Park Iona; *National Park Cameia*), Botswana (Gemsbok), Namibia (**Namib-Naukluft**; Etosha; Skeleton Coast Park)
American black bear (207–526 km^2^): Canada (**Grasslands National Park of Canada**; Moose Mountain Provincial Park; Great Sand Hills; Spruce Woods Provincial Park; Bob Creek Wildland; *Cypress Hills*)
Andean black bear (116–5701 km^2^): Colombia (Tinigua; La Tatacoa; Serrania De Minas), Panama (**Darién**), Peru (*Pampa Hermosa*), Venezuela, Bolivarian Republic of (Cerro Saroche)
Asiatic black bear (1276–23 358 km^2^): India (Hemis), Malaysia (Taman Negara; Endau Rompin (Johor)), Pakistan (Khunjerab), Tajikistan (**Tajik National Park**), Thailand (*Thung Salaeng Luang*)
brown bear (5029–9049 km^2^): Canada (Asatiwisipe Aki Traditional Use Planning Area), Mongolia (**Khangai nuruu**; Har Us Nuur; Tarvagatai nuruu; *Eastern Mongolian Steppe*), Turkmenistan (Kaplangurskiy)
brown hyaena (146–10 494 km^2^): Angola (**National Park Luengue-Luiana**; National Park Quiçama), Botswana (*Kasane*), Namibia (Mudumu; Nkasa Rupara), Zambia (Sioma Ngwezi)
cheetah (6590–22 860 km^2^): Angola (National Park Quiçama), Democratic Republic of the Congo (*Bomu*), Morocco (Dakhla National Park), Mozambique (**Niassa**), Saudi Arabia (‘Uruq Bani Ma'arid; At-Tubayq)
clouded leopard (1238–1911 km^2^): Thailand (**Doi Phukha**; Doi Luang; Khao Bantad; Tham Phathai; Mae Tuen; *Huai Nam Dang*)
dhole (11 724–53 465 km^2^): Mongolia (**Great Gobi**; Gobi Gurvansaikhan range; Gobiin baga /A/, /B/; Khan Khentii; *Khuvsgul*), Tajikistan (Tajik National Park)
dingo (1040–2758 km^2^): Australia (**Cape Arid**; Grampians; Mungo; Stirling Range; Yathong; *Great Otway*)
Ethiopian wolf (2213–19 887 km^2^): Ethiopia (Gambella; Omo; Yangudi Rassa; *Yabello*), South Sudan (**Boma**; Boma Extension)
Eurasian lynx (773–2590 km^2^): Bulgaria (Rila), Italy (Parco nazionale del Pollino; Parco nazionale del Cilento e Vallo di Diano; Parco nazionale del Gargano; *Parco nazionale della Sila*), Ukraine (**Podolskie Tovtry**)
gray wolf (3665–5527 km^2^): Malaysia (Taman Negara), Myanmar (Lenya; *Tanintharyi National Park*), Sweden (**Vindelfjällen**), Thailand (Thungyai Naresuan), United States of America (Olympic)
jaguar (5416–17 884 km^2^): Argentina (La Payunia), Brazil (**Mapinguari**; Campos Amazônicos; Pacaás Novos; Guaporé; *Rio Novo*)
leopard (3399–16 404 km^2^): Cambodia (*Virachey*), Egypt (Wadi El-Gemal - Hamata), Iran (Islamic Republic of) (Urumieh lake), Mauritania (Banc d'Arguin), Morocco (**Dakhla National Park**), Myanmar (Khakaborazi)
lion (7774–99 331 km^2^): Algeria (**Parc Culturel du Tassili (Illizi)**), Angola (National Park Cameia), Congo (Odzala Kokoua), Morocco (Dakhla National Park), Namibia (Namib-Naukluft), South Sudan (*Badingilo Extension*)
puma (6644–13 627 km^2^): Canada (Wabakimi Provincial Park; Asatiwisipe Aki Traditional Use Planning Area; Algonquin Provincial Park; Spatsizi Plateau Wilderness Park; *Northern Rocky Mountains Park*), Peru (**Cordillera Azul**)
red wolf (29–4187 km^2^): United States of America (**Everglades**; Biscayne; *Collier-Seminole*)
sloth bear (115–1740 km^2^): Bhutan (**Jigme Singye Wangchuck**), India (Sundarban; Mouling), Sri Lanka (Knuckles; Peak Wilderness NR; *Sinharaja National Heritage Wilderness Area*)
snow leopard (2977–18 315 km^2^): Mongolia (**Gobiin baga /A/, /B/**; Khangai nuruu; Zed-Khantai-Buteeliin nuruu; Tarvagatai nuruu; Ulaan Taiga; *Myangan-Ugalzat*)
spotted hyaena (632–11 042 km^2^): Cameroon (Mpem et Djim; *Takamanda*), Democratic Republic of the Congo (**Maiko**; Bomu), Namibia (Ai-Ais Hot Springs), Nigeria (Cross River)
striped hyaena (1027–19 092 km^2^): Central African Republic (**Manovo-Gounda-Saint Floris**), Democratic Republic of the Congo (Kahuzi-Biega), India (Kishtwar), Nepal (Langtang), Rwanda (*Akagera*), Saudi Arabia (‘Uruq Bani Ma'arid)
sun bear (1036–1740 km^2^): Bhutan (**Jigme Singye Wangchuck**; *Royal Manas*), Thailand (Sri Lanna; Thung Salaeng Luang; Khao Bantad; Tham Phathai)
Sunda clouded leopard (192–1486 km^2^): Indonesia (**Muara Kendawangan**; Gunung Nyiut Penrissen; Teluk Kelumpang Selat Laut Selat Sebuku; Teluk Apar; Kepulauan Karimata; *Teluk Pamukan*)
tiger (6315–12 599 km^2^): Kyrgyzstan (*Issyk-Kul*), Mongolia (Altai Tavan range), Russian Federation (**Sinyaya**; Olekminsky; Dzhugdzhursky), Uzbekistan (Ugam-Chatkal)

Similarly, we identified the six largest low footprint regions for each large carnivore (figure 3, electronic supplementary material, table S3). These form a set of 150 regions (median area: 5850 km^2^), with areas ranging from 1200 km^2^ (sloth bear) to 1 222 925 km^2^ (leopard) (electronic supplementary material, table S3). As with the protected areas, these low footprint regions cover all the major regions of the world: Africa (*n* = 45), the Americas (*n* = 38), Asia (excluding southeastern Asia) (*n* = 18), Europe (*n* = 21), Oceania (*n* = 6), and southeastern Asia (*n* = 22) (figure 3, electronic supplementary material, table S3). The regions cover 40 countries, most commonly occurring in the USA (*n* = 14), Russia (*n* = 14), Canada (*n* = 10), China (*n* = 9) and Mauritania (*n* = 8) (electronic supplementary material, table S3). Of the 150 region–large carnivore combinations (six per species; note that each region is specific to a different large carnivore species), 22 (14.7%) would result in an intact carnivore guild following reintroduction of the large carnivore species there (electronic supplementary material, figure S1, table S3). On average, 8.0% of each low footprint region occurs within protected areas, with the lowest averages observed for the spotted hyaena (0.0%), cheetah (0.0%) and lion (0.1%), and the highest averages for the dingo (26.8%), Andean black bear (23.6%) and Sunda clouded leopard (21.4%) (electronic supplementary material, table S3). The average human footprint for these regions was highest for the sloth bear (7.1), clouded leopard (3.9) and Eurasian lynx (3.9) and lowest for the snow leopard, spotted hyaena and cheetah (all 0.0) (electronic supplementary material, figure S1, table S3). The carnivore species with the greatest median (across low footprint regions) numbers of preferred prey species available were the jaguar (*n* = 8), dingo (*n* = 2.5), red wolf (*n* = 2), gray wolf (*n* = 2) and Eurasian lynx (*n* = 2) (electronic supplementary material, table S3).

Species-specific maps of the six largest protected areas and low footprint regions reveal several spatial patterns (electronic supplementary material, figure S2). Compared to protected areas, low footprint regions are often clustered together (e.g. for the lion and leopard) as human footprint is strongly spatially autocorrelated (electronic supplementary material, figure S2). For species with lost ranges covering parts of Northern Africa, large low footprint regions nearly always occur there (as human footprint is low in this area of the world), while protected areas tend to be more evenly distributed (because there are relatively few large protected areas in Northern Africa) (electronic supplementary material, figure S2).

**Figure 5.**
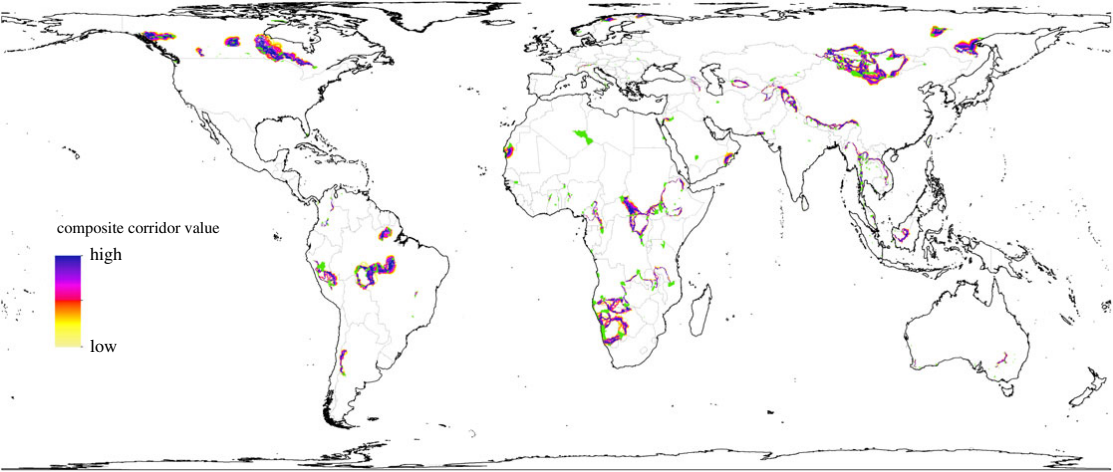
Corridors among the 411 protected areas that we identified as candidate sites for large carnivore reintroduction based on the 25 largest protected areas for each large carnivore species. The protected areas are shown in green. Corridors between protected areas were identified using Linkage Mapper and are coloured according to their value based on compositing normalized cost-weighted distance rasters, with higher composite corridor values corresponding to greater potential contributions to connectivity. We used the human footprint map (linearly rescaled to range from 1 to 100) as the resistance raster for calculating cost distances, with areas containing cropland masked out. A 500 km Euclidean distance threshold was used to avoid mapping corridors between protected areas that are more than 500 km apart. Note that zooming can be used to view detail in this figure.

The species with candidate protected areas that have the greatest connectivity (as measured by mean distance to nearest protected area) were the clouded leopard (0 km), sun bear (0 km), dingo (2 km) and puma (2 km), while the species with the least protected area connectivity were the lion (223 km), cheetah (179 km), striped hyaena (116 km) and leopard (115 km) (electronic supplementary material, table S2). In terms of corridor availability, large groups of connected protected areas were observed in much of the world, with regions of particularly high concentration including Mongolia, Central and Southern Africa and Canada (figure 5).

## 4. Discussion

### 4.1. Protected area analysis

The protected area analysis highlights protected areas for each large carnivore species where the likelihood of reintroduction success may be high. The smallest of the six largest protected areas is substantially larger than the estimated critical reserve size that is required for 50% probability of persistence for all ten of the large carnivore species with critical reserve size estimates [18]. Additionally, there are many other protected areas within the lost ranges of these species that exceed the species’ estimated critical reserve sizes (electronic supplementary material, figure S3). Even relatively small protected areas may be viable options for reintroductions as part of broader-scale programmes (e.g. systems of linked protected areas) or with active predator management techniques like fencing [33]. Regardless of protected area size, excessive human activities within or near reserve boundaries can make it difficult for large carnivores to persist, although there are exceptions such as Asiatic lions in the Gir Forest National Park of India [34]. Our finding of relatively high (≥10.0) average human footprint in protected areas for the Eurasian lynx, sloth bear and Asiatic black bear suggests that this may be a particularly serious issue for these species, although recent large carnivore recoveries in Europe indicate that carnivore persistence in heavily modified landscapes is possible in certain cases provided that prey are abundant and persecution is limited [12]. As the lynx has had little range contraction in Asia, its lost range occurs nearly entirely within Europe, where human impacts have been relatively high [9]. Similarly, the sloth bear and Asiatic black bear have lost ranges in south Asia and southeastern Asia where anthropogenic impacts are common. This relates to the problem posed by ‘paper parks’ where human encroachment into the park (in the forms of, for example, bushmeat hunting and habitat loss) renders the protected area ‘protected’ in name only [35,36]. Thus, reintroduction attempts in protected areas must take into account the actual level of protection. This is particularly true in West and Central Africa, where subsistence and commercial hunting commonly occur in protected areas [37]. Given the sensitivity of large carnivores to both direct hunting and to loss of prey base, continued efforts to map bushmeat hunting pressure (using predictors like distance to roads and human population density) are vital to ensuring appropriate selection of reintroduction areas [8,38]. Such broad-scale mapping and modelling efforts would need to be followed up with local ground-truthing to either verify that reintroduction is a practical option or to determine what intermediate steps are required prior to reintroduction. Possible steps to reduce bushmeat hunting pressure include strengthening legal protections for wild mammals, providing alternative food sources to humans and increasing access to education and family planning [39].

The focus of our analysis is on identifying broad spatial patterns in potential reintroduction areas, rather than making detailed arguments in support of any particular reintroduction effort. We now briefly discuss a few specific possibilities that are particularly intriguing. Following the extirpation of gray wolves from Olympic National Park in the early 1900s, elk have had strong negative impacts on riparian plant communities, which has been linked to erosion of river banks [40]. This trophic cascade suggests that Olympic National Park, the fifth largest protected area that we identified for the gray wolf, should be considered as a candidate site for reintroducing this species. Another possible repatriation is the red wolf to Everglades National Park. This reintroduction was considered several decades ago by the Red Wolf Recovery Team [41]. Additionally, it was recently the subject of an online petition, which noted that red wolves may help control the introduced Burmese python population in Everglades National Park as their diet includes snakes and snake eggs [42]. These examples, and many others, illustrate the possibility of the re-establishment of important ecological effects following large carnivore reintroductions. The literature on large carnivores and trophic cascades suggests that such potential ecological impacts are likely to be common [7,43]. Effects could be particularly strong within protected areas because human impacts (e.g. agriculture) often do not play a dominant role in protected reserves [44]. While the reintroductions that we consider correspond to spatial scales much smaller than those of species' historic ranges, they could lead to range expansions that significantly increase the spatial extent of large carnivores’ ecological effects.

### 4.2. Low footprint region analysis

The low footprint regions in our analysis may be considered as candidate areas for reintroduction either immediately or following the establishment of additional protected areas there. Many other low footprint regions (beyond the six largest for each species) may also be well-suited for reintroduction (electronic supplementary material, figure S4). By looking at regions with low human footprint, we partially avoid the issue of paper parks because the footprint map is based on more objective criteria. On the other hand, some of the most serious threats to large carnivores like bushmeat hunting (of both the carnivores and their prey) are difficult to map globally, and are probably not adequately reflected in the human footprint map or any other existing global map [39]. For example, many of the potential reintroduction sites in this portion of our analysis are located in the Sahara-Sahel region of North Africa (electronic supplementary material, figure S2). Species with sites in this region include the African wild dog, cheetah, leopard, lion and spotted hyaena (electronic supplementary material, figure S2). Although this region tends to have low human footprint, reintroduction may not be practical here. Threats to biodiversity in this area include climate change, the availability of firearms used for hunting, overgrazing and other forms of habitat loss, widespread extraction of natural resources, water overexploitation and extreme political instability [45].

Connectivity must also be considered when interpreting the low footprint region results. We defined these regions as contiguous areas with low human footprint at 5 km grid cell resolution. Often, this resulted in adjacent regions separated by only a narrow band of grid cells, corresponding to a road. For example, see regions two and three for the African wild dog (electronic supplementary material, figure S2). The extent to which such adjacent regions should be treated as separate depends on the nature of the road or other division as well as the biology of the large carnivore species. In general, it seems reasonable that roads often divide low footprint regions as they have well-documented negative effects on many large carnivores including being associated with vehicle collisions, aggressive carnivore–human encounters, hunting and habitat loss and fragmentation [46]. Habitat fragmentation associated with roads (suggesting region isolation) has been documented for gray wolves, black bears, brown bears, Andean black bears and pumas [46]. Further study of connectivity in this context could be useful to determine the likelihood of passive expansions, wherein carnivores naturally return to parts of their former ranges. When practical, passive expansions may be preferred to active reintroductions as they often require less human involvement and wildlife handling.

### 4.3. Limitations

Our analysis has several key limitations. First, there is uncertainty associated with the data sources that we used. Reconstructing historic range maps is difficult, and our historic map set only provides rough approximations to species' historic ranges [9]. These correspond to roughly AD 1500 and thus our analysis reflects a relatively recent baseline time period [47]. There is also uncertainty associated with the current range map set. Additionally, range maps do not provide information on species abundances, making it impractical to assess where translocations could bolster existing populations and to determine baseline predator densities required for ecological effectiveness, which could then be used to set targets for reintroduced populations [48]. The inaccuracies in our range map set imply that some of the proposed sites for each large carnivore species may either presently contain or have never contained the species. For example, several of the proposed sites for the African wild dog may be too dry to have supported this species and some of the northernmost proposed sites for the brown hyaena may lie beyond the extent of its true historic range. Although we attempted to mitigate this issue by manually checking the status of the carnivore species–protected area combinations, this was not practical for the latter problem (determining whether or not the protected area ever contained the large carnivore species). In many cases, we were also not able to conclusively determine whether or not the protected area currently contains the large carnivore species.

Another class of limitations is associated with our use of protected areas to explore candidate sites for rewilding. While we confined our search to strictly protected areas (IUCN categories I–III) to reduce the likelihood of mistakenly identifying insufficiently protected areas, in practice, the extent to which these areas are protected varies greatly and may not be fully reflected in the human footprint map [36]. In some parts of the world, categories I–III protected areas may not be suitable for reintroductions, while in other parts, even category IV (Habitat/Species Management Area), V (Protected Landscape/Seascape) or VI (Protected Area with sustainable use of natural resources) protected areas (which we did not consider) could be appropriate targets for reintroductions. Other protected area-specific variables that may be relevant include management budget, government versus private ownership, and whether or not hunting is allowed [49]. Even when the actual level of protection is high, without appropriate buffer zones, policies in nearby areas can negatively affect carnivore populations. For instance, hunting and trapping of gray wolves in areas near Denali and Yellowstone National Parks may impact populations within these parks [50–52]. A closely related limitation applies to the map of protected area connectivity in that habitat quality may be inadequate along some of the mapped corridors (figure 5).

Although not necessarily a limitation, portions of our analysis are probably sensitive to our use of 5 km resolution. While higher resolution could be beneficial in certain cases, it might be inappropriate for identifying contiguous low footprint regions. In any case, the actual resolution of global range maps, especially those for historic ranges, is often of the order of several degrees or lower, making our choice of resolution adequate for our global analysis [53].

A final limitation of our analysis is that we were not able to explicitly order protected areas and low footprint regions by reintroduction potential. Although we present results ordered by protected area (or low footprint region) size, which is a key predictor of reintroduction success, we do not claim that this is the only predictor worth considering. Rather, we also include data on average human footprint, prey availability (when such data exist), protected area category (for protected areas), portion of the region protected (for low footprint regions), etc. (electronic supplementary material, tables S2 and S3). Our hope is that these data and results can serve as both a global view of rewilding possibilities and as a preliminary guide to more targeted carnivore reintroduction programmes. All results that we present need to be more carefully validated when possible, especially if they are to be interpreted at local scales. This is critically important in the context of human tolerance for and policy towards large carnivores, which we could not quantify in our analysis, but are known to be vital to the survival of these species [54,55]. For example, control of dingoes is legally mandated throughout New South Wales, Australia, which means that dingo reintroductions are not practical there [56]. It is also important that managers quantify prey abundance and demographics prior to reintroduction, rather than simply looking at the number of prey species present [2]. When preferred prey species are found to be absent in an area, reintroducing these species first can help to ensure predator reintroduction success, although some have argued that it is better for prey and predator reintroductions to occur together rather than sequentially [57]. The potential for strong effects of predation on naive prey means that initial prey abundances may need to be relatively high in order to buffer prey declines while antipredator behaviour is regained [58,59].

### 4.4. Conservation implications

Our study is the first to provide a spatially explicit global assessment of future large carnivore rewilding possibilities. Although some have avoided using the term ‘rewilding’ in situations where species are reintroduced to landscapes that have been modified by humans [60], we embrace this term because rewilding need not require the exclusion of all human activity and large carnivores can help to maintain ecosystem processes, which are closely linked to the idea of wildness [61]. Additionally, following predator reintroduction, formerly naive prey populations can exhibit increased vigilance and antipredator behaviour within a single generation [17]. Other indicators of wildness include solitude and remoteness, both of which may be linked to protected areas and, especially, to regions with low human footprint [62]. Ultimately, predators like gray wolves are often seen as symbols of wildness, blurring the distinction between reintroduction and rewilding [63,64].

As mentioned in the limitations section, all protected areas and low footprint regions identified in our analysis must be thoroughly validated before any reintroduction attempts. This would need to include assessment of prey base in and around the region, connectivity with other reserves, hunting pressure, potential for human–carnivore conflict, funding available for reintroduction programmes, relevant laws and regulations, extent of habitat loss, and other anthropogenic pressures [8,39]. It is important that the original causes of extirpation be understood and, if necessary, mitigated prior to any reintroduction attempts [65]. This is especially critical for sites near the current ranges of species, because the causes of extirpation may be preventing natural recoveries there. Our analysis is probably overly optimistic in that many threats to wildlife are difficult to quantify and may be severely underestimated at the global scale. It is possible that for some of the large carnivore species, none of the candidate sites that we identified are appropriate for reintroductions. For instance, regions with political instability and ongoing military conflicts can encompass large areas where reintroduction is not practical [66]. When planned reintroductions or natural recoveries in the near future are not realistic, broad-scale public-policy reforms can improve prospects for large carnivore conservation in the longer term. Not only can such transformative policies and actions help to increase long-term opportunities for successful large carnivore reintroductions, they can, in some cases, decrease the necessity of reintroductions by making natural returns more likely.

While we focused on individual areas in our analysis, larger scale connectivity is also essential to large carnivore survival. This is particularly true in Africa where many protected areas are relatively isolated and quickly becoming more isolated in response to rapid human population growth [67]. Large carnivores tend to have extensive home ranges, and may frequently roam outside even the largest protected areas [60]. Thus, ensuring that the areas around reintroduction sites have suitable habitat and high human tolerance for carnivores will help to promote reintroduced population survival. Many of the protected areas that we highlight are near other, smaller protected areas that could help sustain predator populations (electronic supplementary material, table S2). Additionally, the numerous corridors that we mapped among the 25 largest protected areas in each species' lost range (411 protected areas in total) suggest that reintroduced carnivores may be able to naturally traverse to other candidate reintroduction areas (figure 5). This suggests that reintroduction into unfenced reserves may have the greatest long-term likelihood of success, but there are possible drawbacks. For instance, African lion densities tend to be closer to estimated carrying capacities in fenced reserves than in unfenced reserves, probably due to fenced reserves having reduced human–lion conflict (e.g. over livestock depredation), habitat loss and poaching [49]. Fenced reserves can also benefit humans by reducing the risks posed by large carnivores. On the other hand, it has been argued that African lion populations in fenced reserves tend to be small and that fencing leads to fragmentation, limits dispersal routes, and leads to genetic isolation [68]. This is especially problematic for reintroductions as they often begin with small founding populations that may lack genetic diversity [2]. In any case, the extent to which fenced reserves should be prioritized for rewilding depends greatly on the nature of human activities in the surrounding habitat and should be considered on a case-by-case basis. This issue is less applicable in poorer countries where fencing large protected areas may be infeasible given management budgets.

A less commonly considered benefit of reintroductions is derived from viewing reintroductions as landscape-scale natural experiments conducted through time. The ecological knowledge that can be gained from such broad-scale interventions is invaluable, particularly given that the lack of documented trophic cascades for many large carnivore species could be due to their small current population sizes and a shortage of appropriate data [7,69]. Predator reintroductions have already led to innovative work in the areas of community assembly, alternative stable states, and behavioural mediation in trophic cascades [21,70]. The potential for future work is rich, ranging from disentangling the ecological effects of top predators from other factors using causal modelling to further studying the effects of reintroduced predators on naive prey, which is important as many prey species are themselves endangered and predation on naive prey poses ethical and animal welfare challenges [15,47,71].

Together with the extensive literature on successful carnivore reintroduction efforts (see introduction), our analysis illustrates the potential for global rewilding of the Earth's large carnivores across their former ranges. While care must be taken to mitigate risks to humans, reintroducing large carnivores can be a major part of carnivore conservation, promote functioning ecosystems, benefit numerous other species and provide wildlife viewing opportunities for humans [16,17]. The decision to promote large carnivore rewilding is consistent with a desire to strengthen ecosystem services that benefit humans. Moreover, it is a way of acknowledging the intrinsic value of these species, by helping them to once again flourish across their former ranges [71]. While the present endangerment of many large carnivore species has an array of complex multifaceted causes, the ultimate driver is human-derived (anthropogenic) pressures. Similarly, conserving many of these species may require bold human intervention, without which, they may eventually be committed to extinction in the wild [72]. Thus, if carnivore conservation and all its associated benefits are a priority, carefully planned rewilding efforts will almost certainly be necessary as part of broader conservation programmes.

## Supplementary Material

supplement (Supplementary Figures and Tables)

## Supplementary Material

SI_tables (Additional Supplementary Tables)

## Data accessibility

In addition to the publicly available data sources that we cite in the text, relevant data are given in electronic supplementary material, tables S2 and S3.
