# Correction to ‘Rewilding the world's large carnivores’

**DOI:** 10.1098/rsos.180514

**Published:** 2018-05-02

**Authors:** Christopher Wolf, William J. Ripple

*R. Soc. open sci.* **5**, 172235. (Published 14 March 2018). (doi:10.1098/rsos.172235)

## 

In the second row of fig. 1, the second photograph from the left was incorrectly described in the caption as showing a cheetah (*Acinonyx jubatus*). It is actually a photograph of a leopard (*Panthera pardus*).

