# Supplementary material for: Rewilding the world's large carnivores
Source: R Soc Open Sci. 2018 Mar 14;5(3):172235. doi: 10.1098/rsos.172235 (PMC5882739; doi:10.1098/rsos.172235)
Supplement: supplement (Supplementary Figures and Tables) [file rsos172235supp1.docx]

**Supporting Figures and Tables Legends**

**Table S1 (Excel file).** Photo credits for the images used in Figure 1.

**Table S2 (Excel file).** The 25 largest category I-III protected areas in the ‘lost’ (historic minus current) ranges of each large carnivore species. The columns are: ‘Species’ (species’ common name), ‘Protected area’ (protected area name), ‘IUCN Category’ (protected area category indicating level of protection), ‘Area (km^2^)’ (terrestrial area of the protected area), ‘Footprint’ (mean human footprint across the protected area), ‘% in low footprint’ (percentage of protected area within the bottom 10% of species’ lost range for human footprint), ‘Nearest PA (km)’ (distance to nearest category I-III protected area), ‘Country’ (primary country for the protected area), ‘Guild’ (if true, rewilding the species there would result in a complete large carnivore guild), ‘Prey’ (preferred prey species of the predator that are present in the protected area), ‘Number of prey’ (number of preferred prey species present in the protected area), ‘Total prey’ (total number of preferred prey species identified for the large carnivore), ‘Status’ (if available, specifies the status of the large carnivore in the protected area – i.e., whether it is present, absent, or its status is unknown; the top six largest PAs with status ‘absent’ or ‘unknown’ are highlighted in green), ‘Source’ (the source website for the status data), and ‘Status notes’ (notes, if any, for the status entry).

**Table S3 (Excel file).** The 25 largest contiguous low (bottom 10% within lost range) human footprint regions in each carnivore species’ lost range. Variables descriptions are given in Table S2 with the exceptions of ‘Region ID’ (the area rank of the region) and ‘% Protected’ (the percentage of the region overlapping category I-III protected areas). ‘Country’ is based on the country that contains the greatest area of the low footprint region.


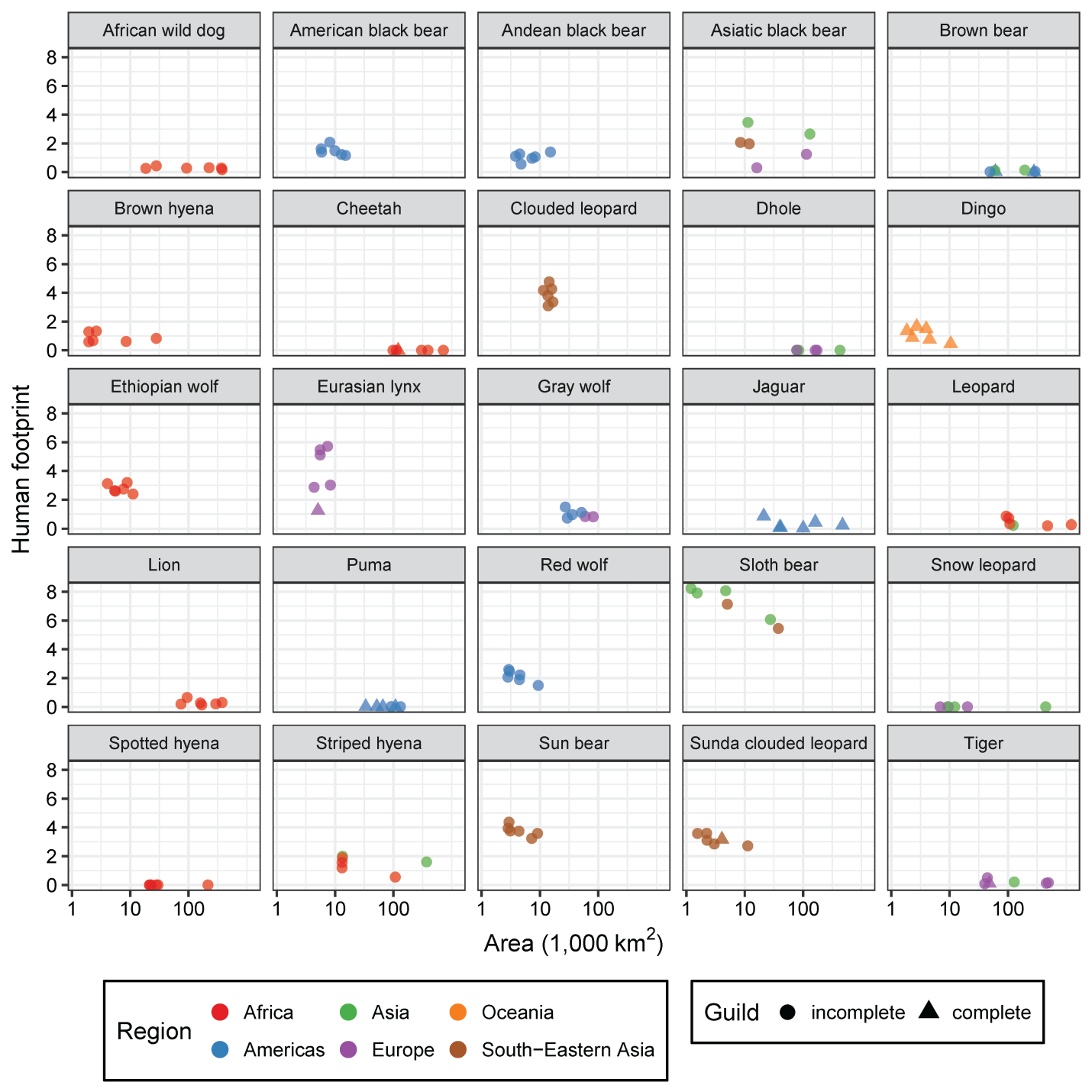


**Figure S1.** The six largest contiguous low footprint regions inside the ‘lost’ (historic minus current) ranges of each large carnivore species. Low footprint regions were defined using the bottom 10% threshold for each species. For each carnivore species, variables shown are the mean human footprint across the protected area, the region of the world, and whether or not the large carnivore guild becomes complete following reintroduction of the carnivore species.

**Figure (set) S2.** Potential sites for reintroducing large carnivores. The first panel in each figure shows the six largest strictly protected areas within the large carnivore species’ lost range (i.e. where the species has been extirpated). The second panel shows the six largest low footprint regions within the lost range of the species. Low footprint regions were determined based on contiguous regions within the ‘last of the wild’ regions for each large carnivore species’ lost range. ‘Last of the wild’ regions are those in the bottom 10% for human footprint within each species’ lost range. Legends correspond to both low footprint regions (region IDs are given before the semicolons) and protected areas (names are given after the semicolons). In both cases, the areas are sorted by geographic area from largest to smallest. For example, for the African wild dog, the largest protected area (shown in red; left map) is Namib-Naukluft and the largest low footprint region (also shown in red; right map) is region number 1. Circles are drawn around small areas to make them more visible.**
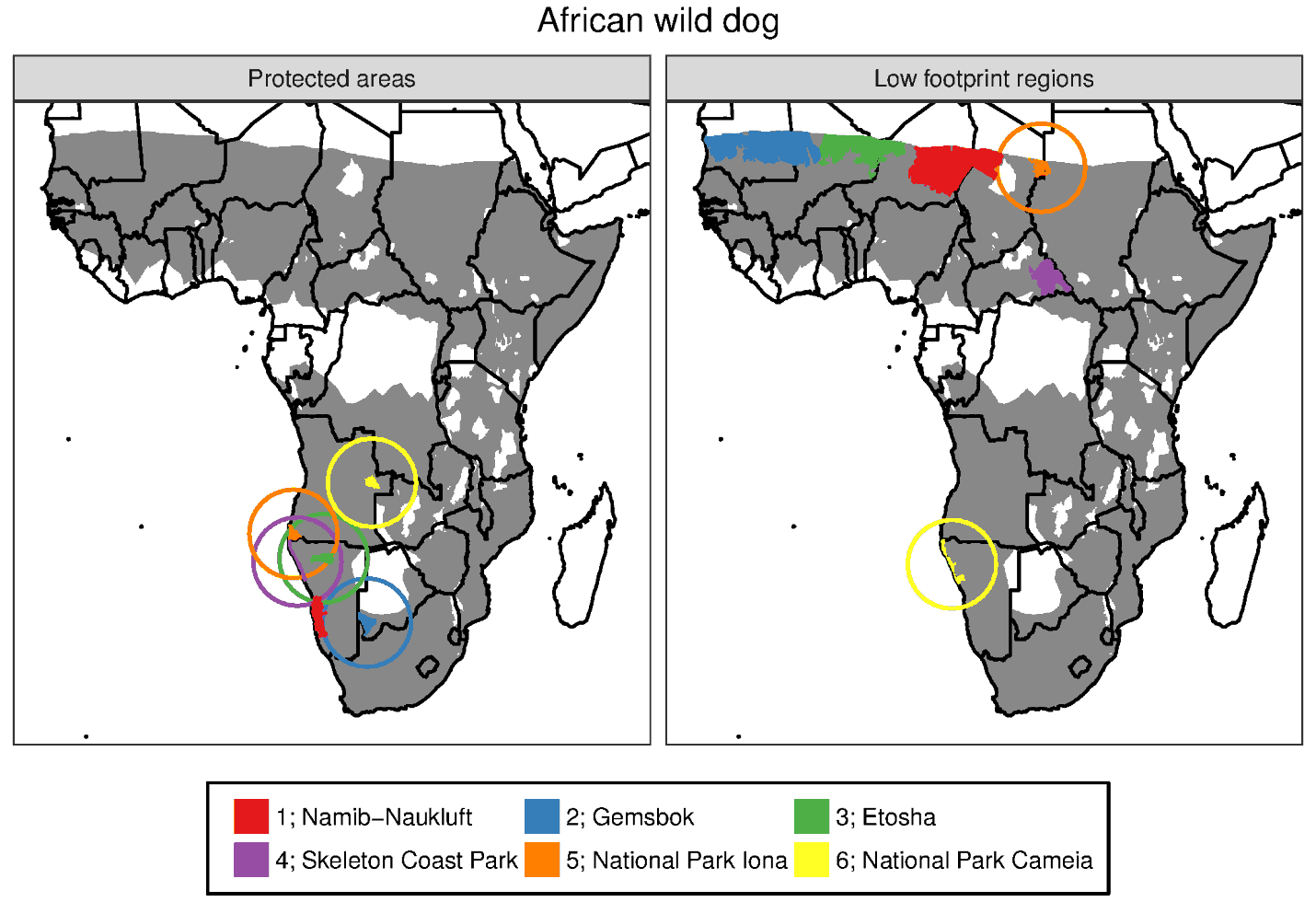

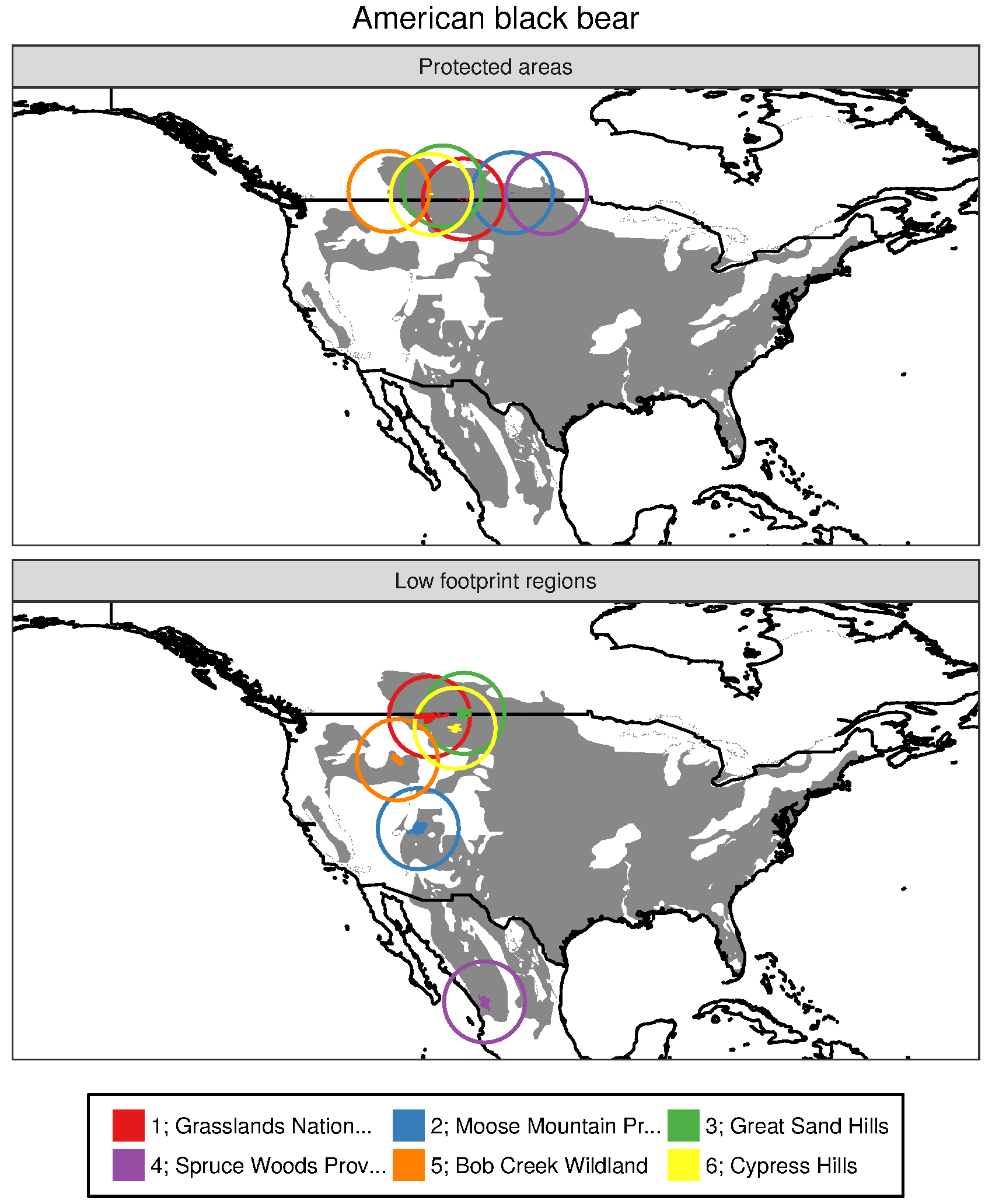

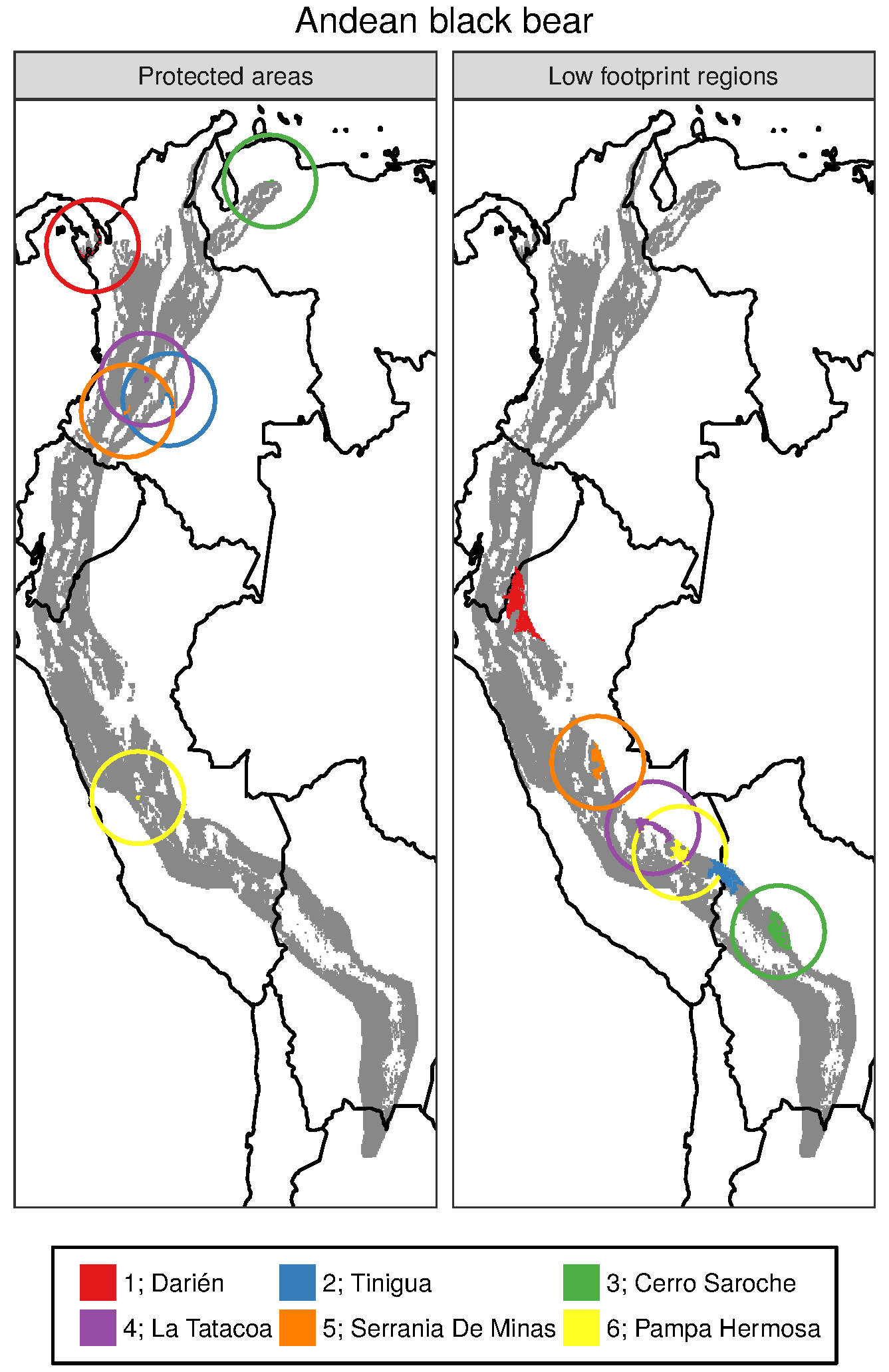

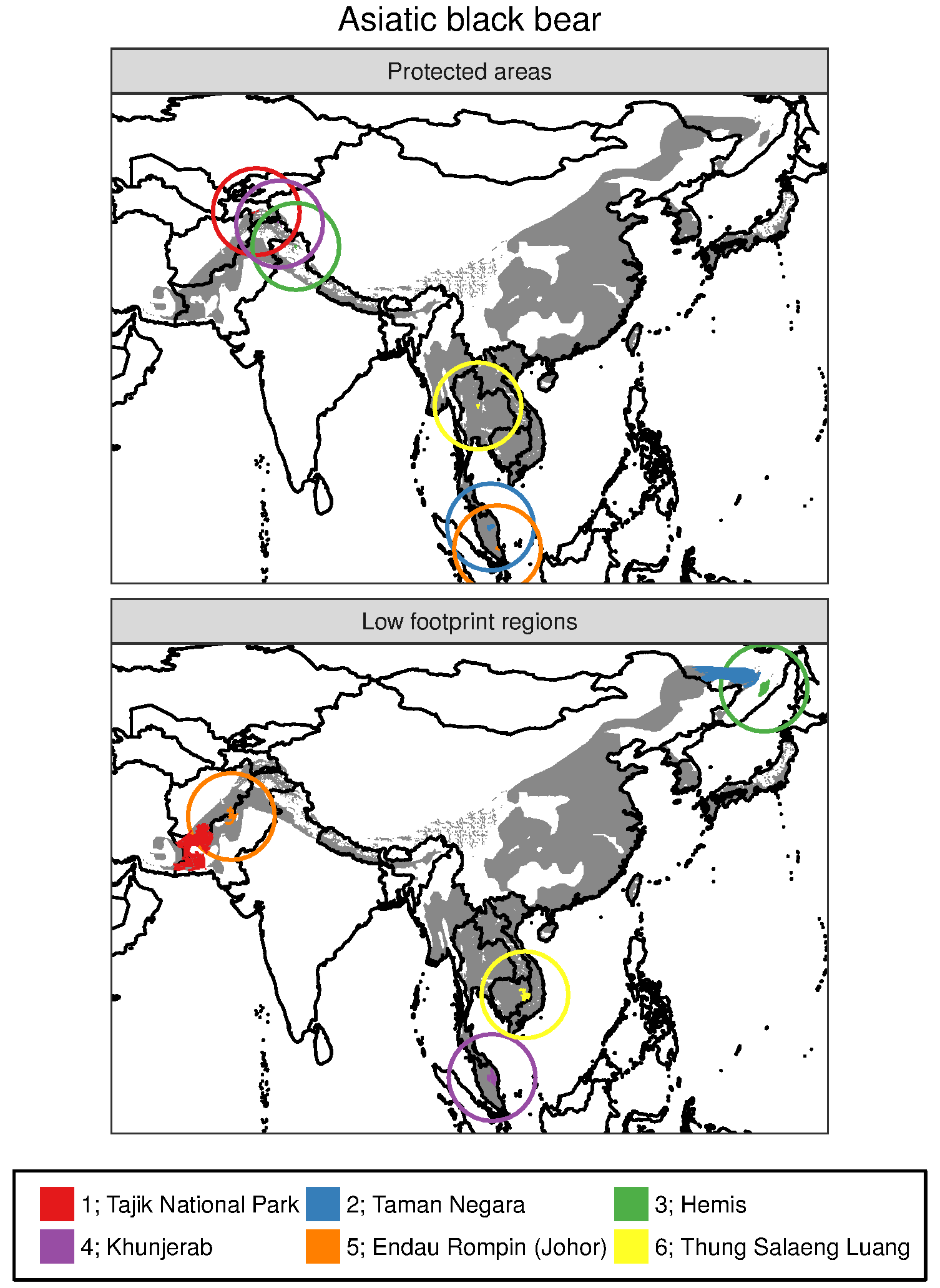

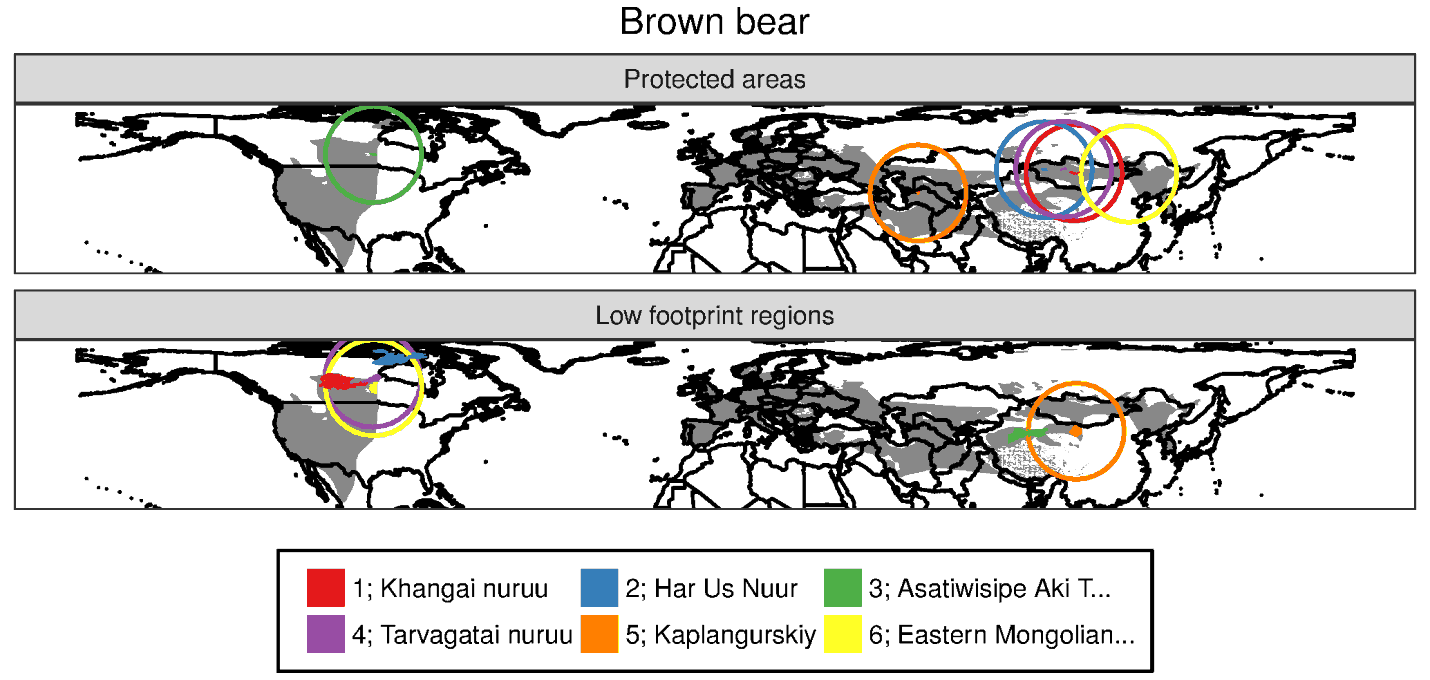

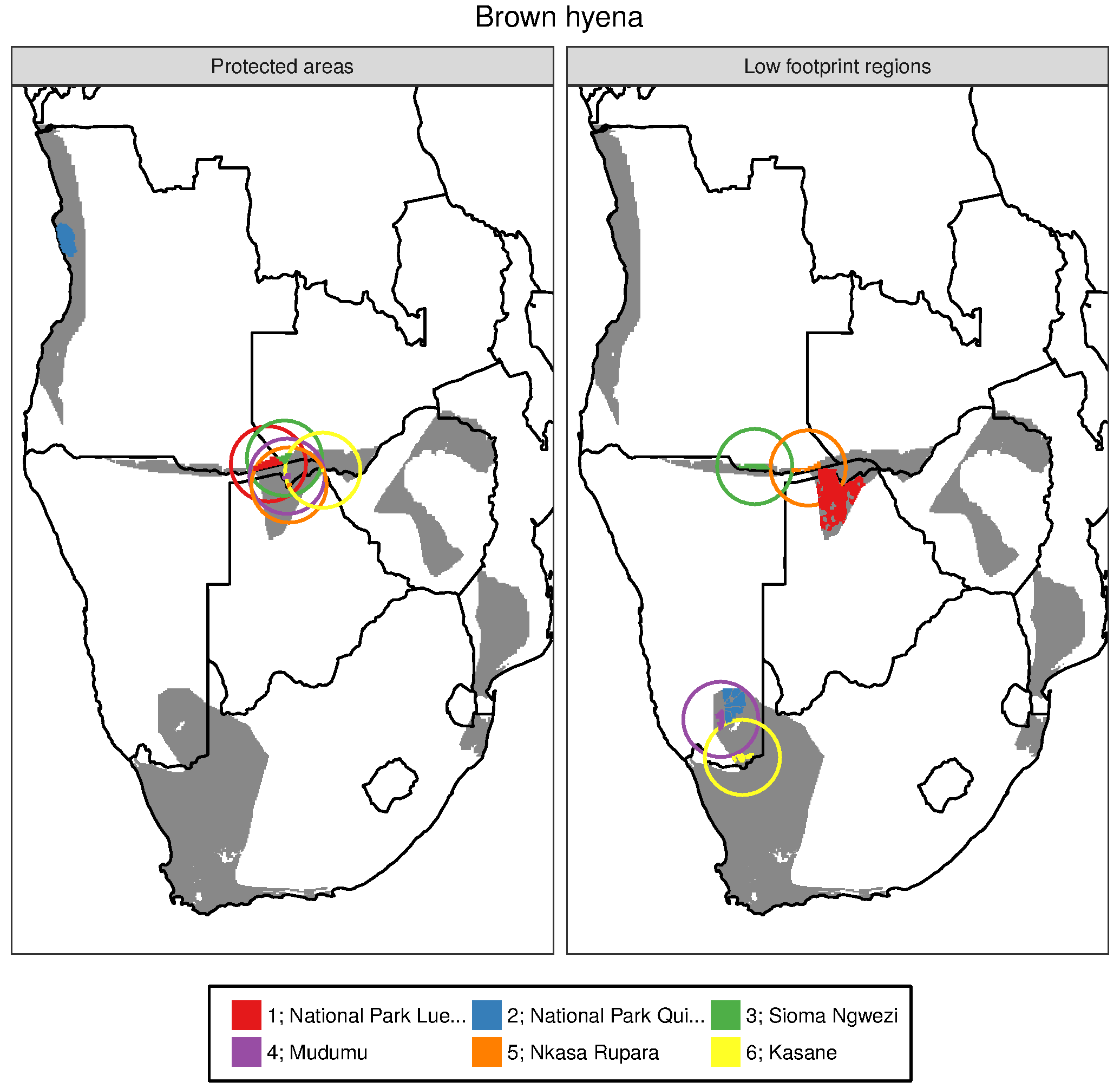

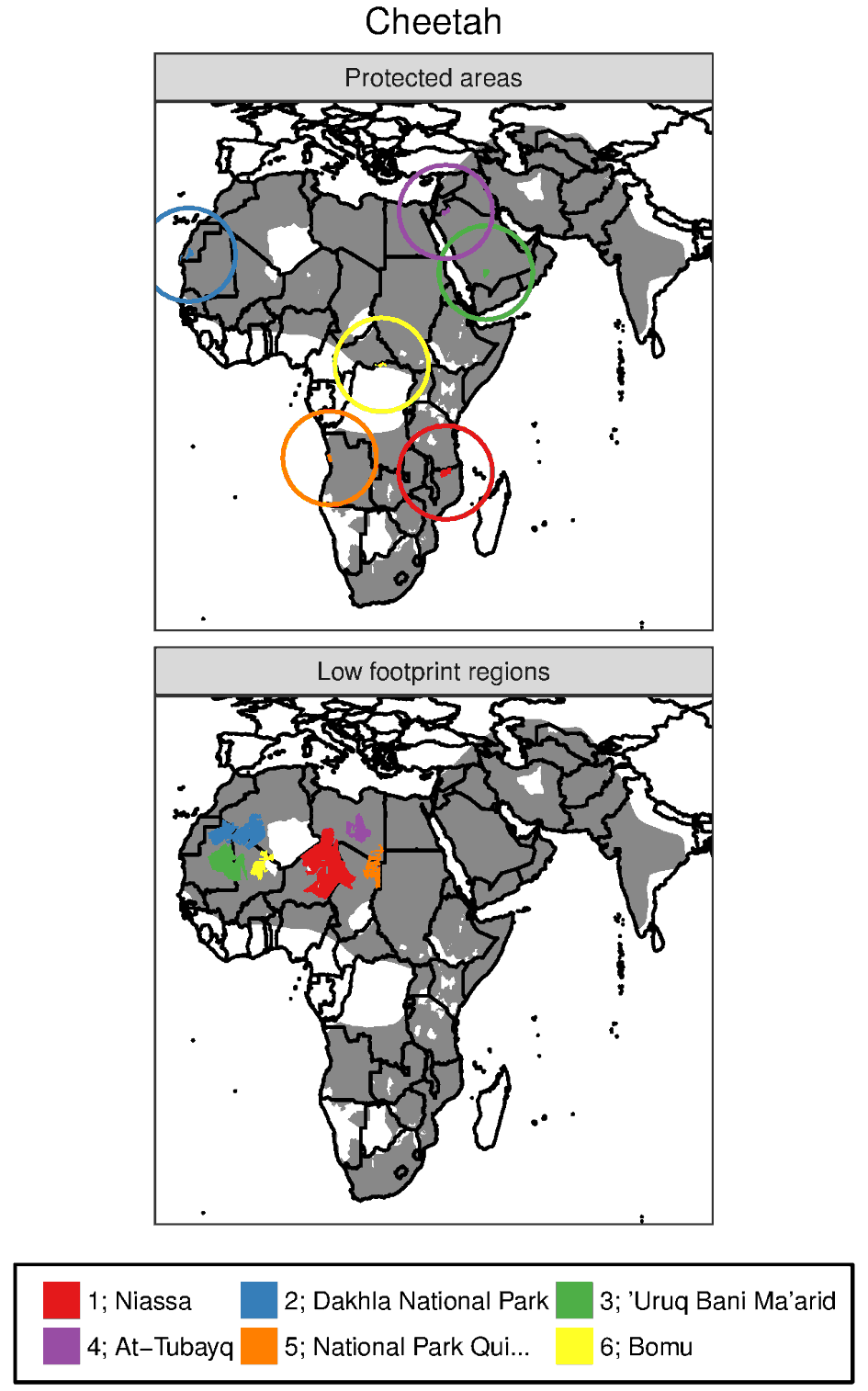

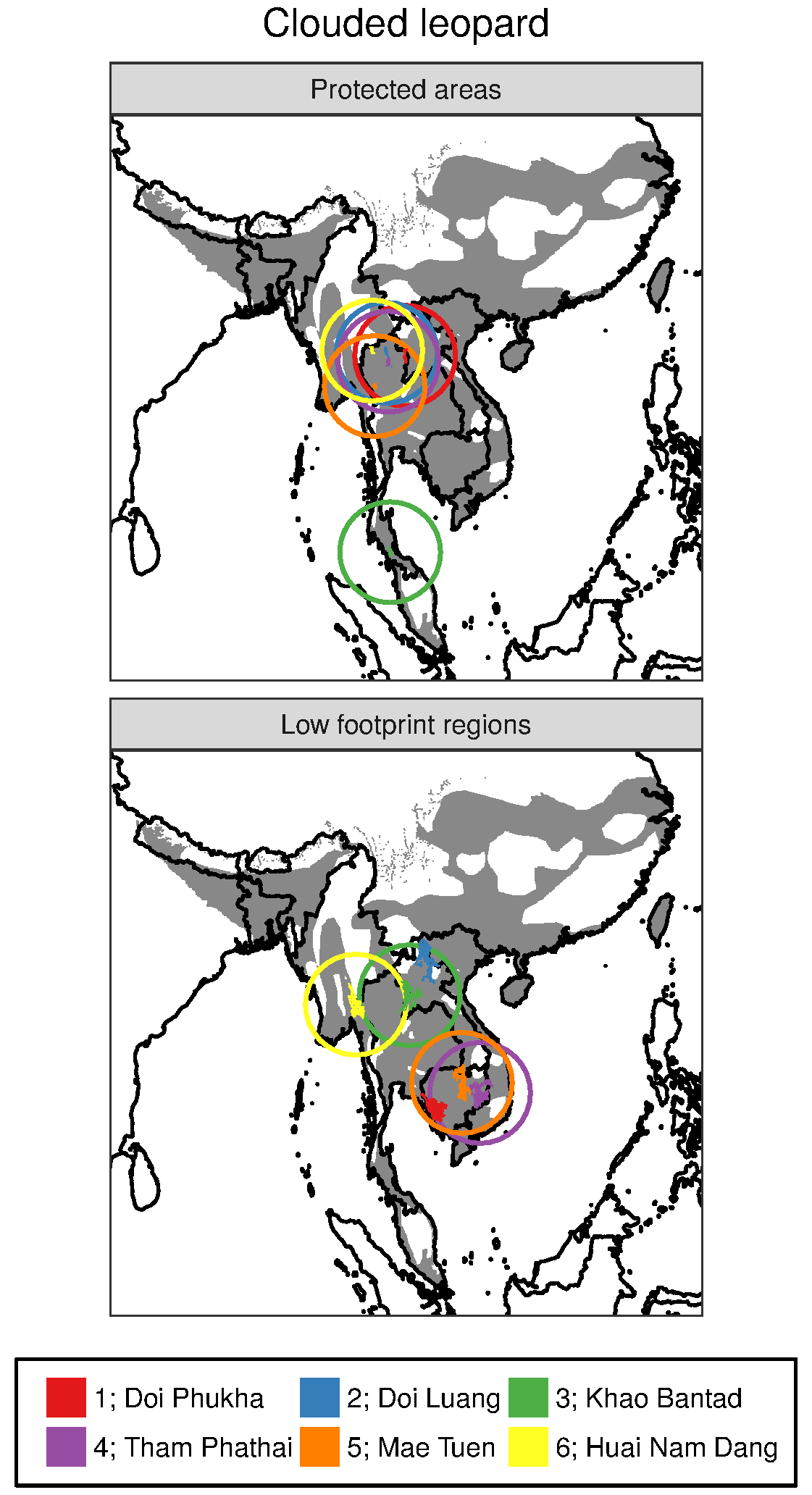

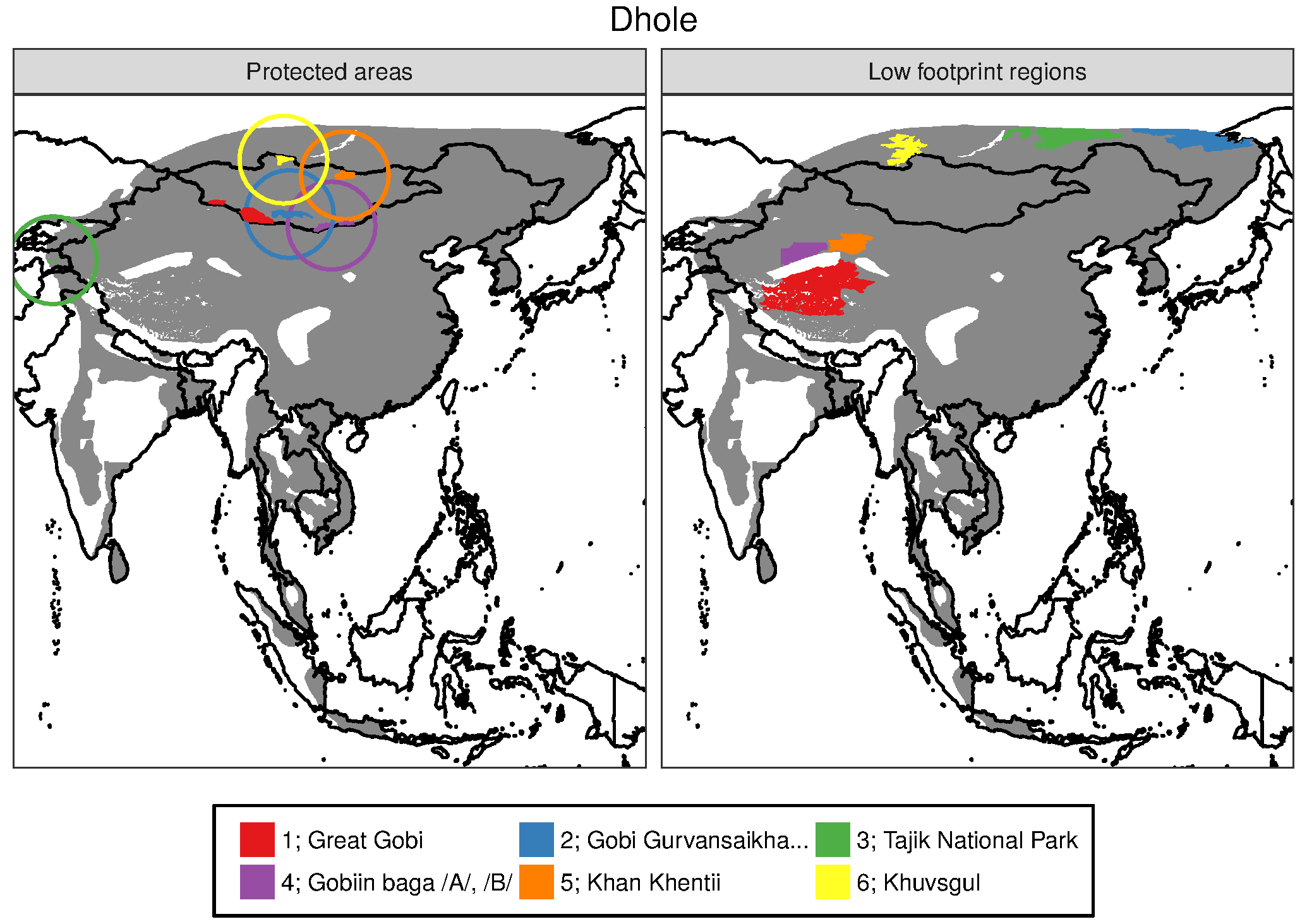

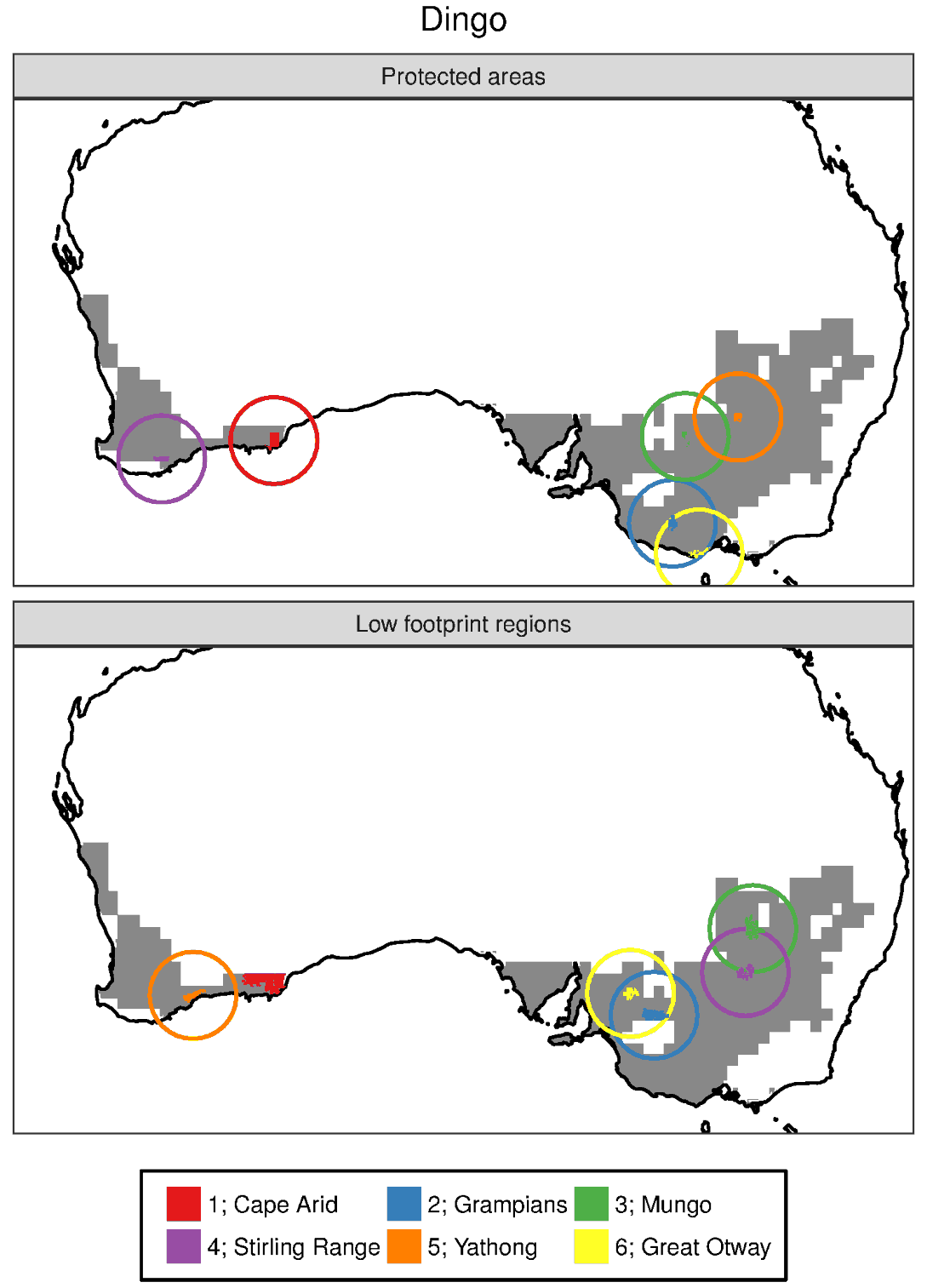

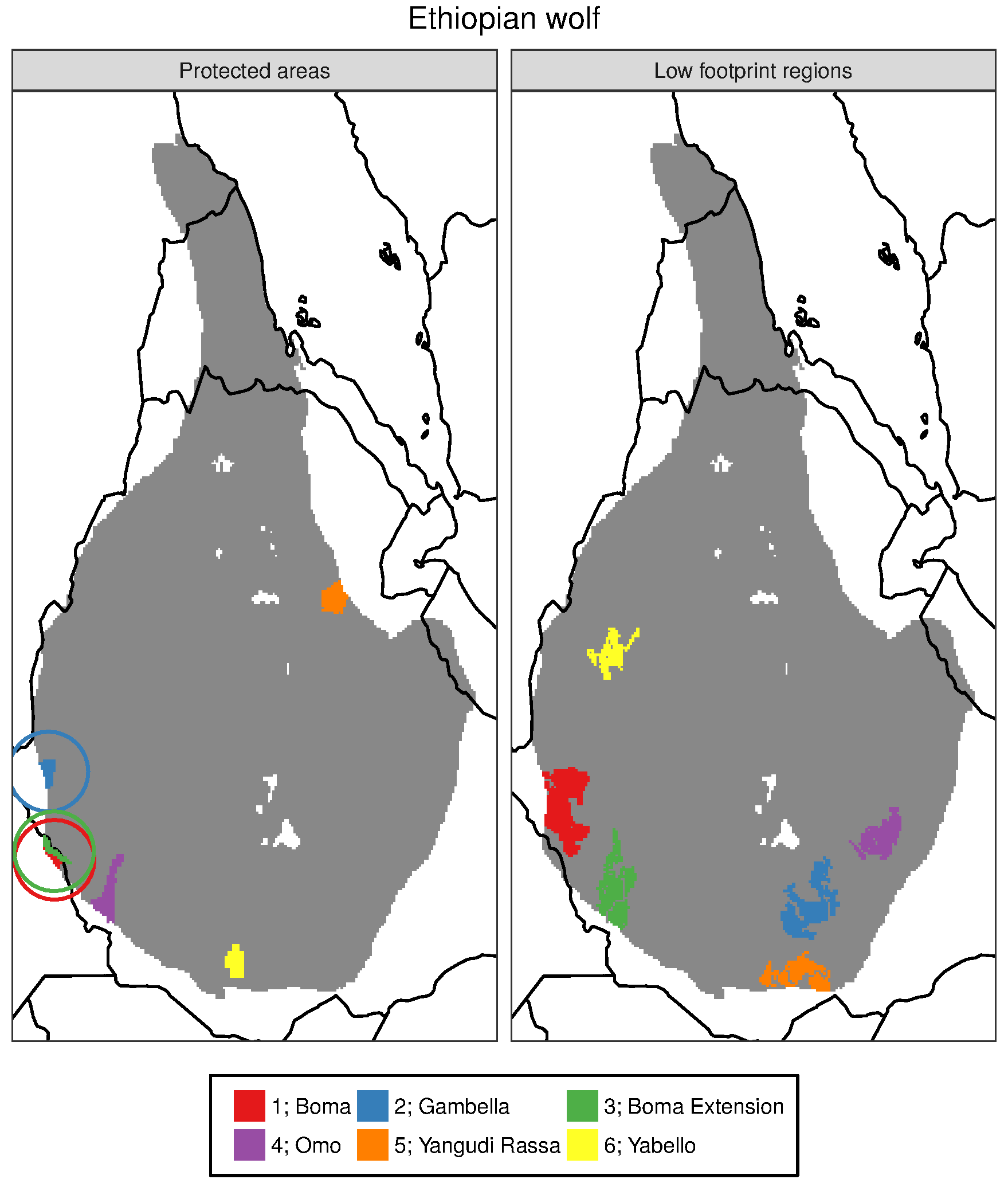

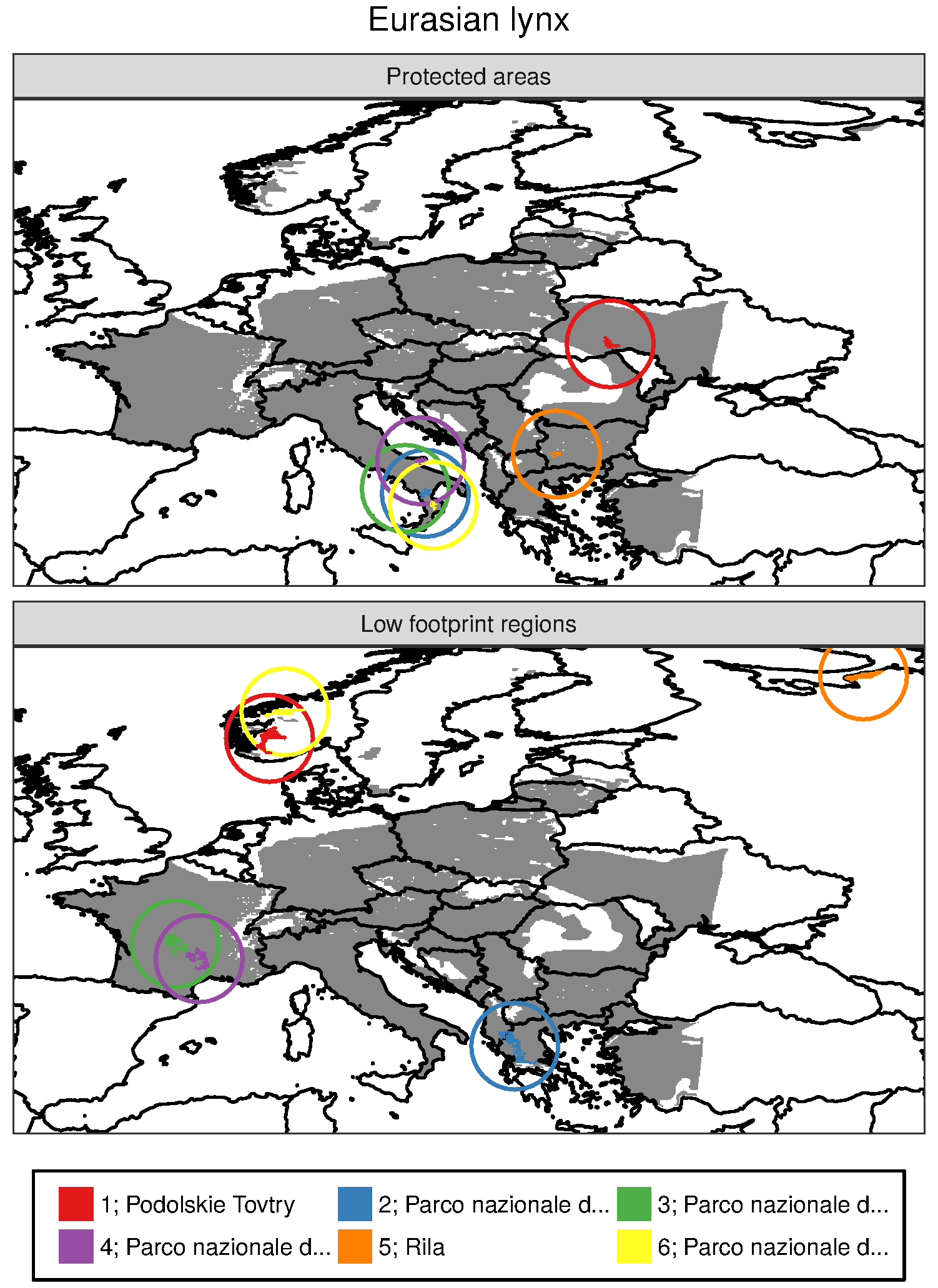

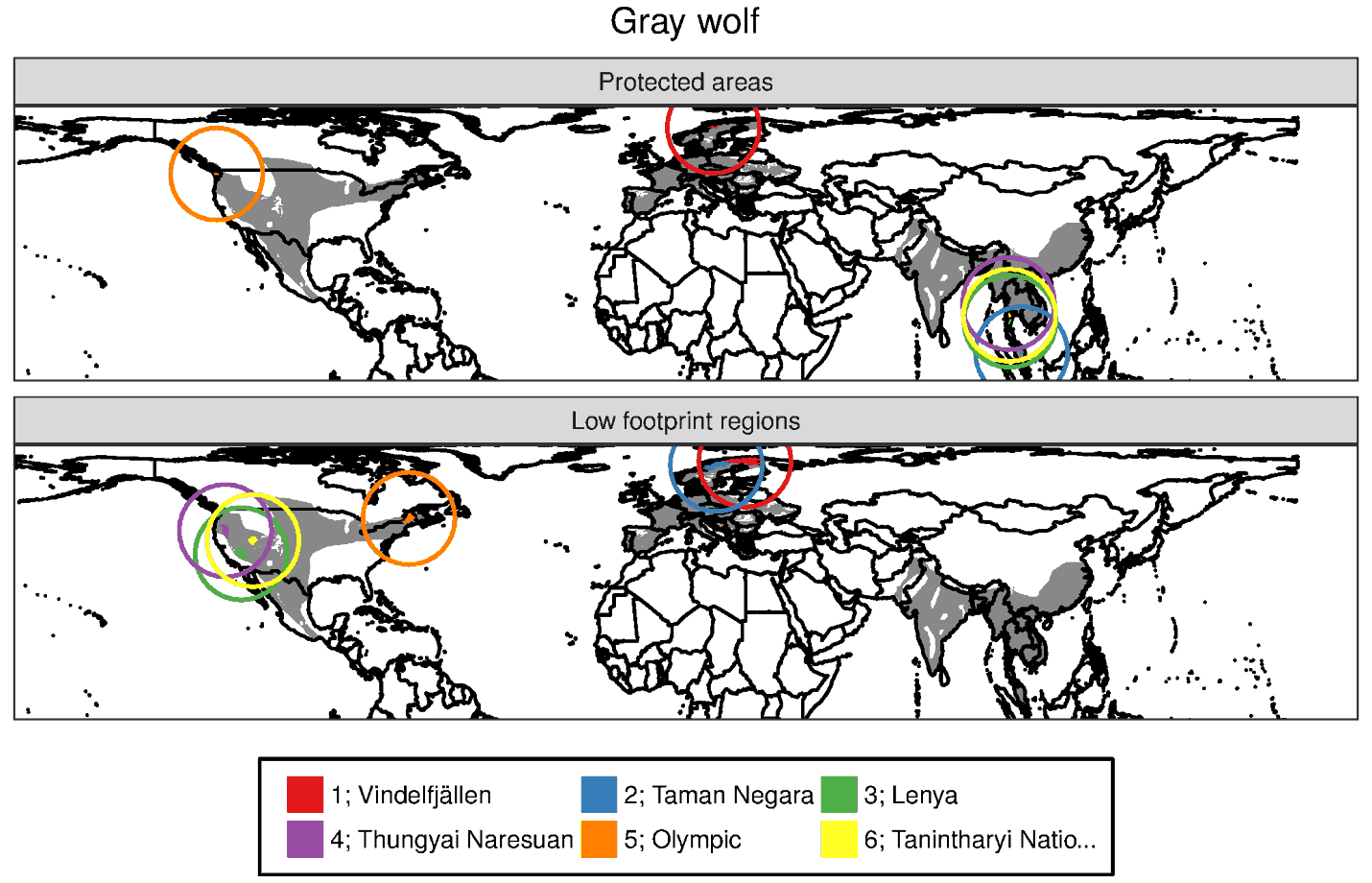

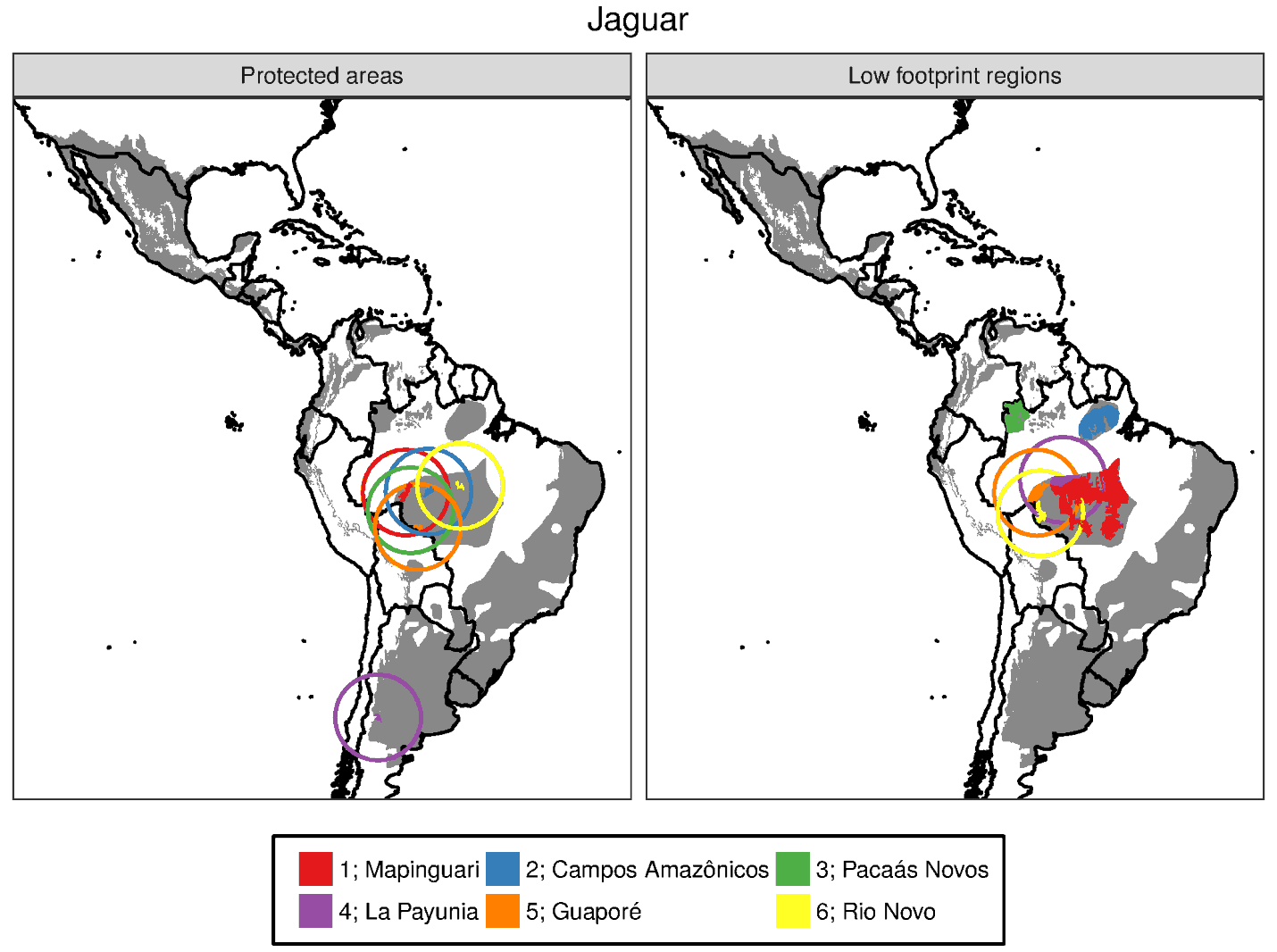

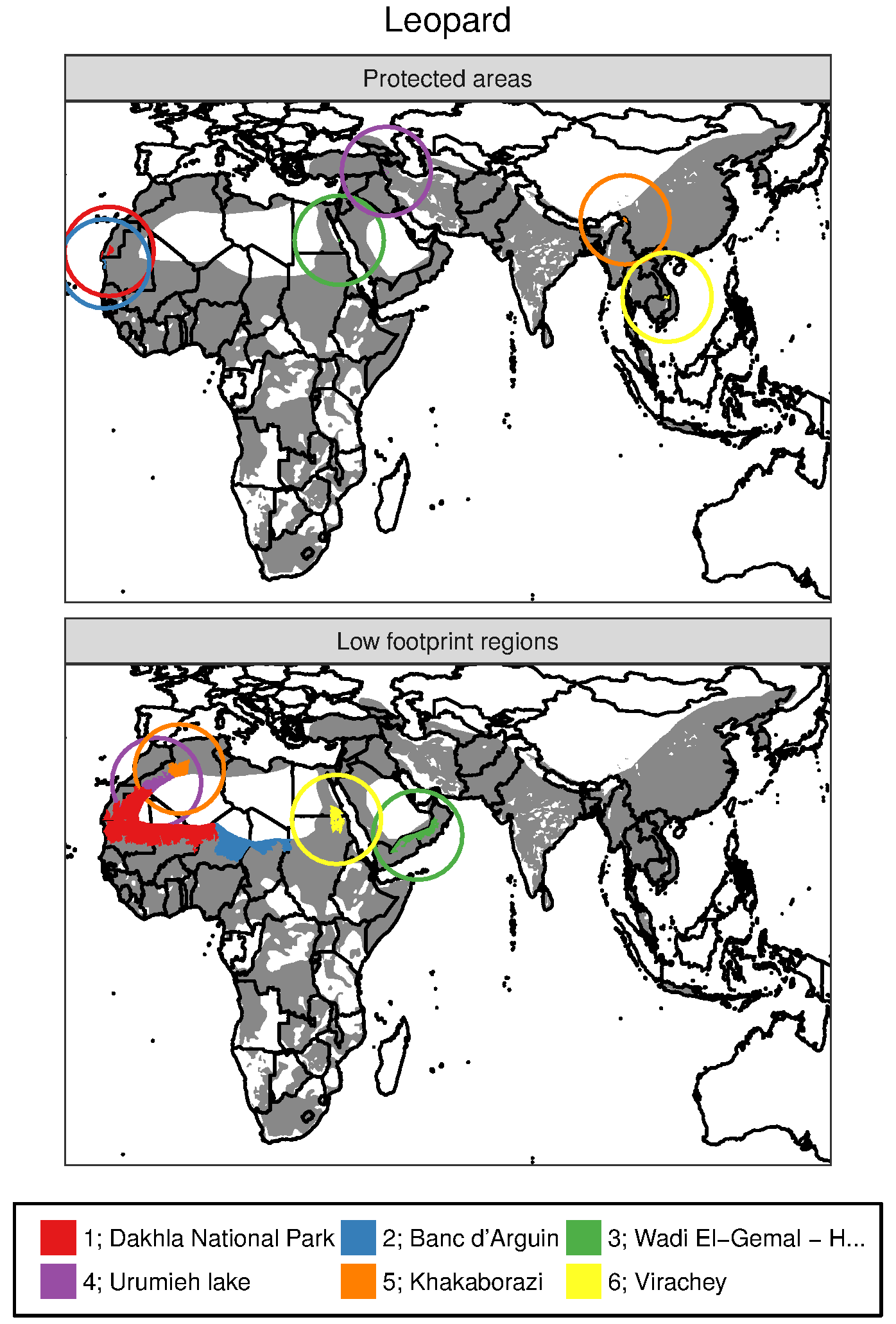

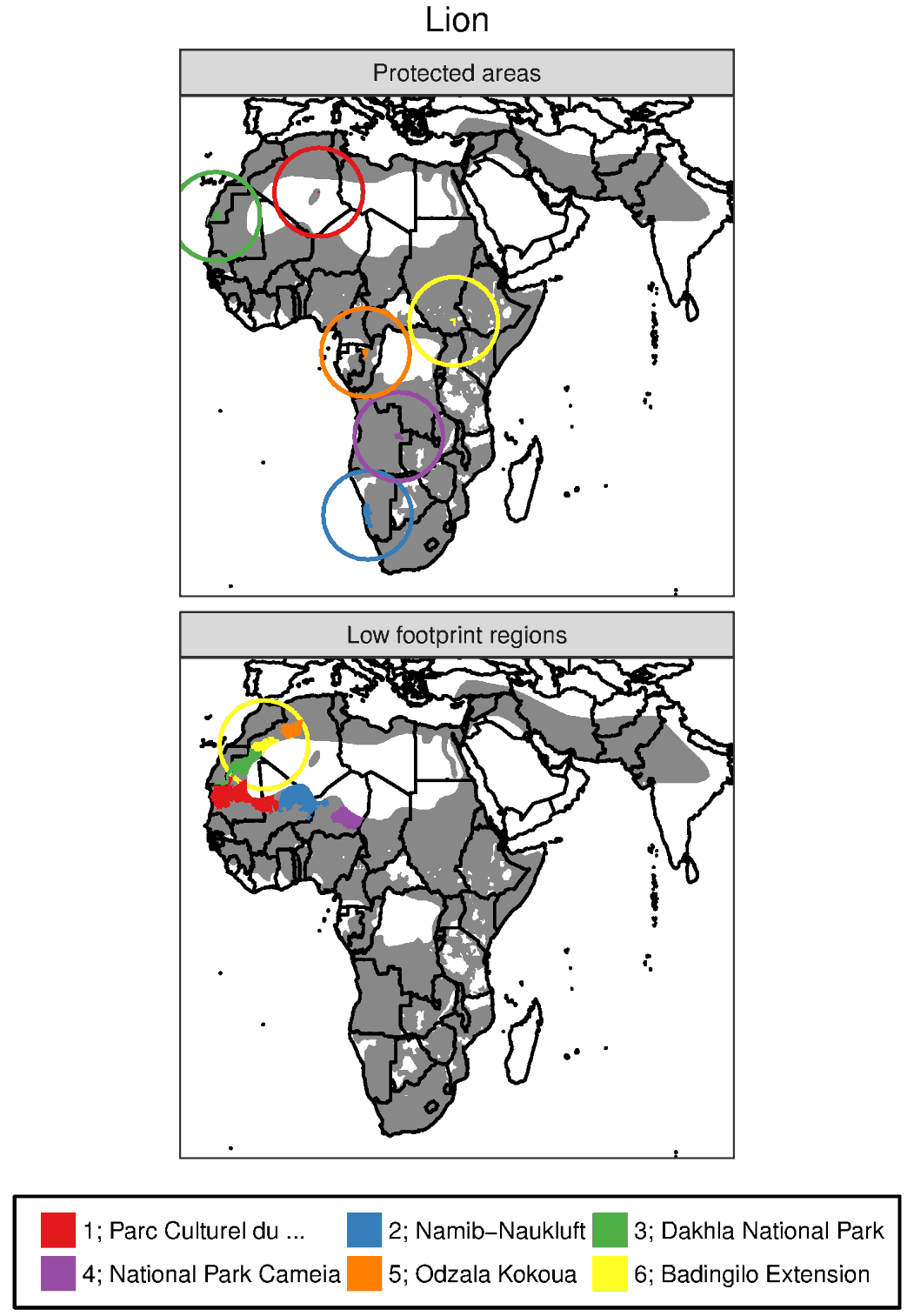

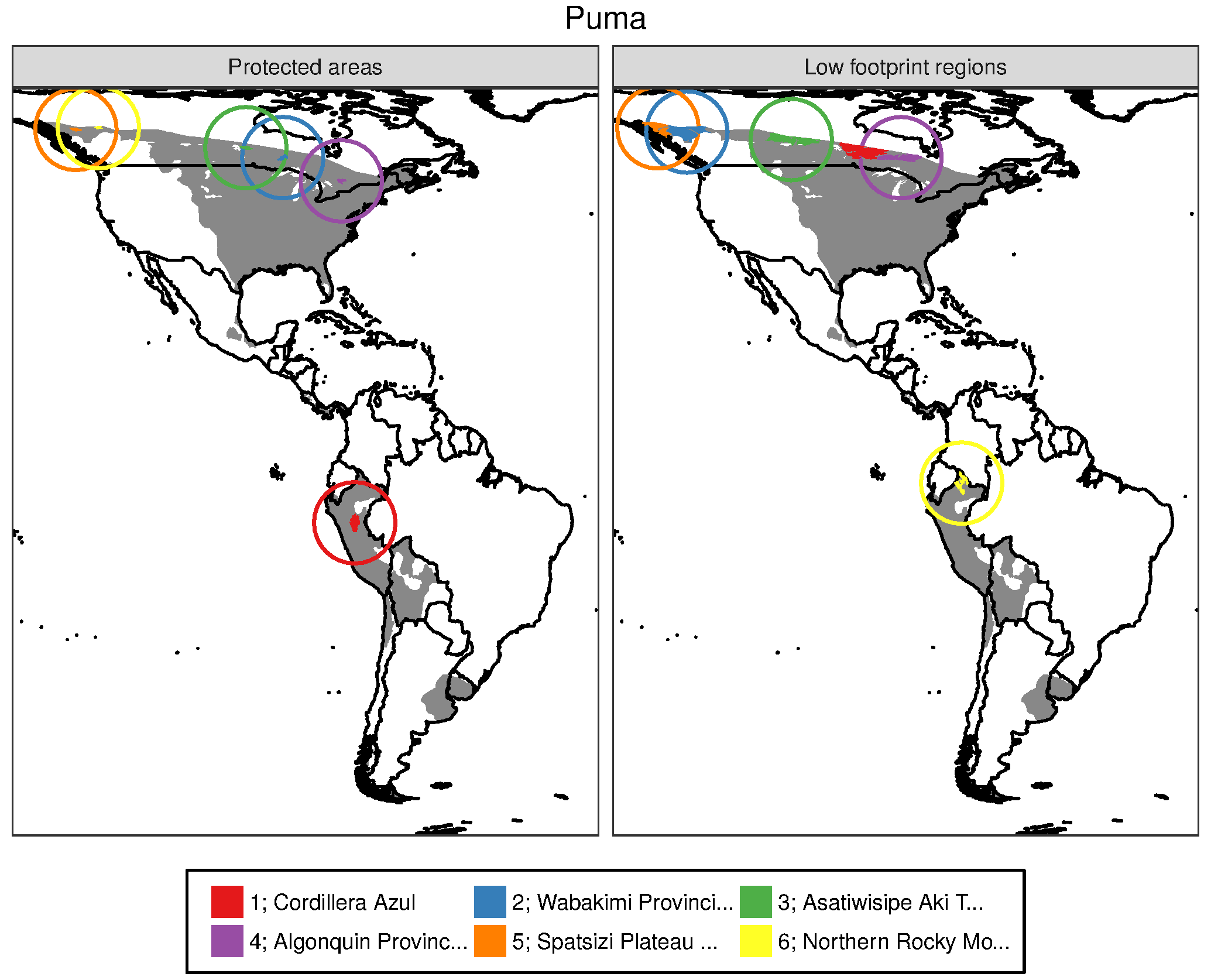

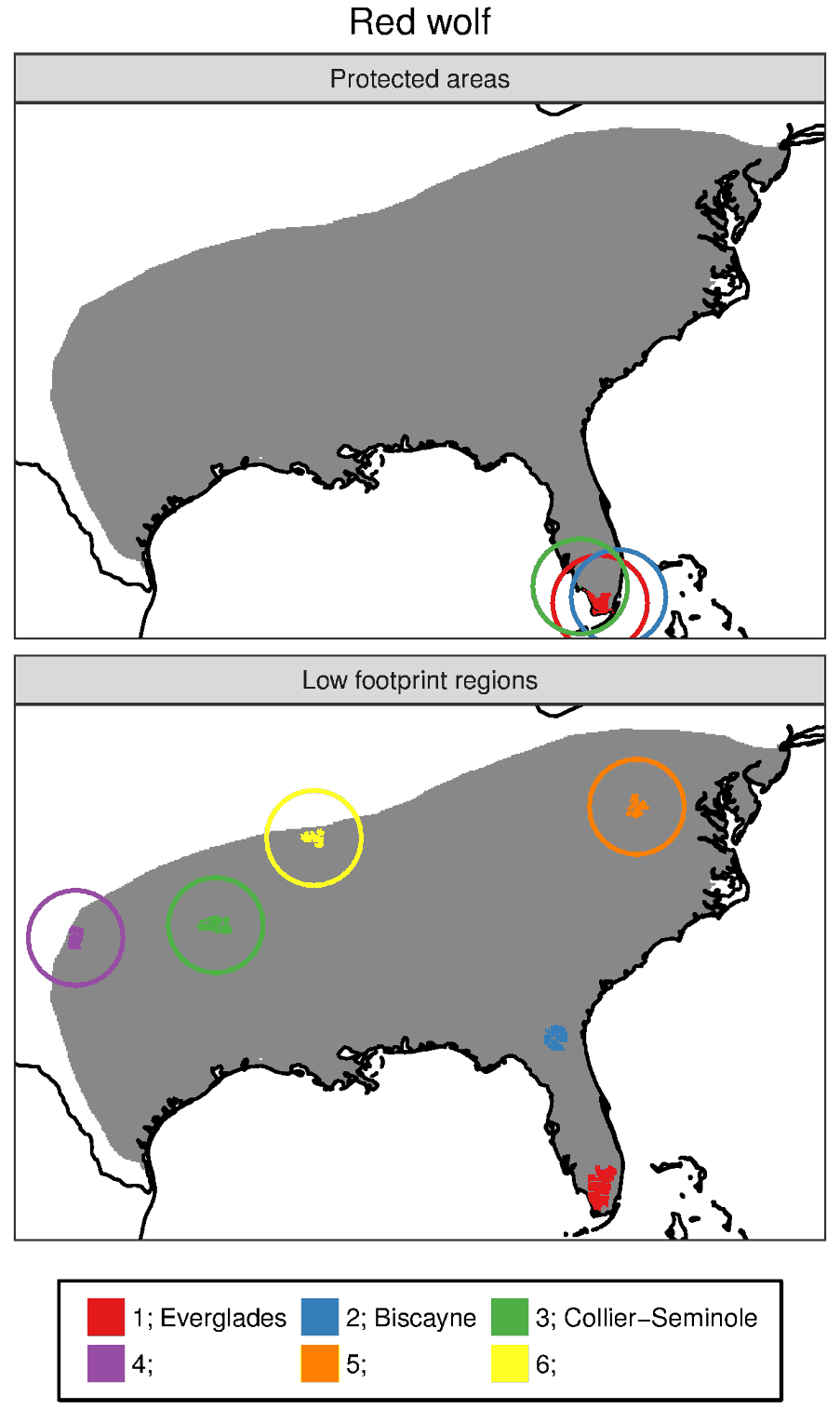

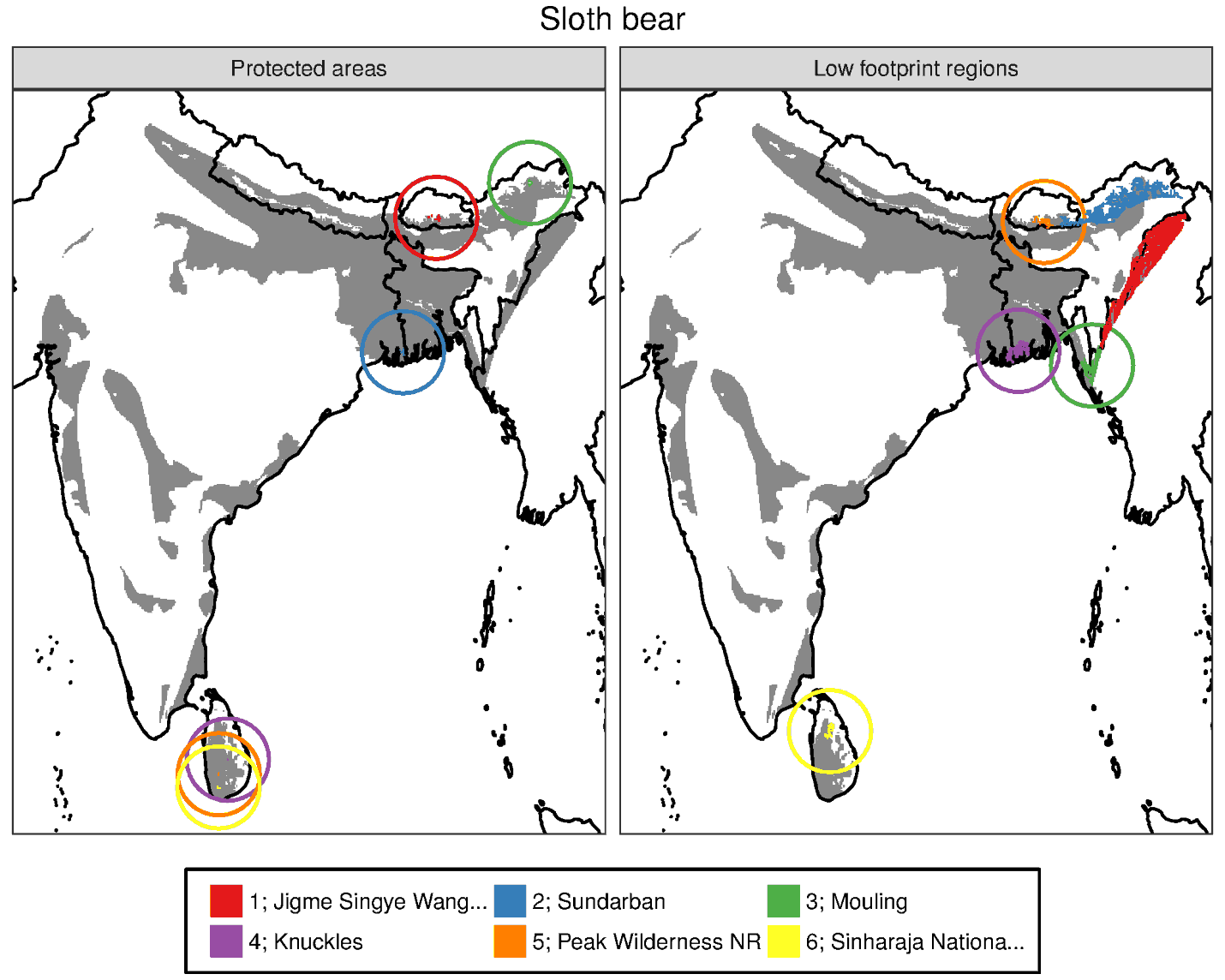

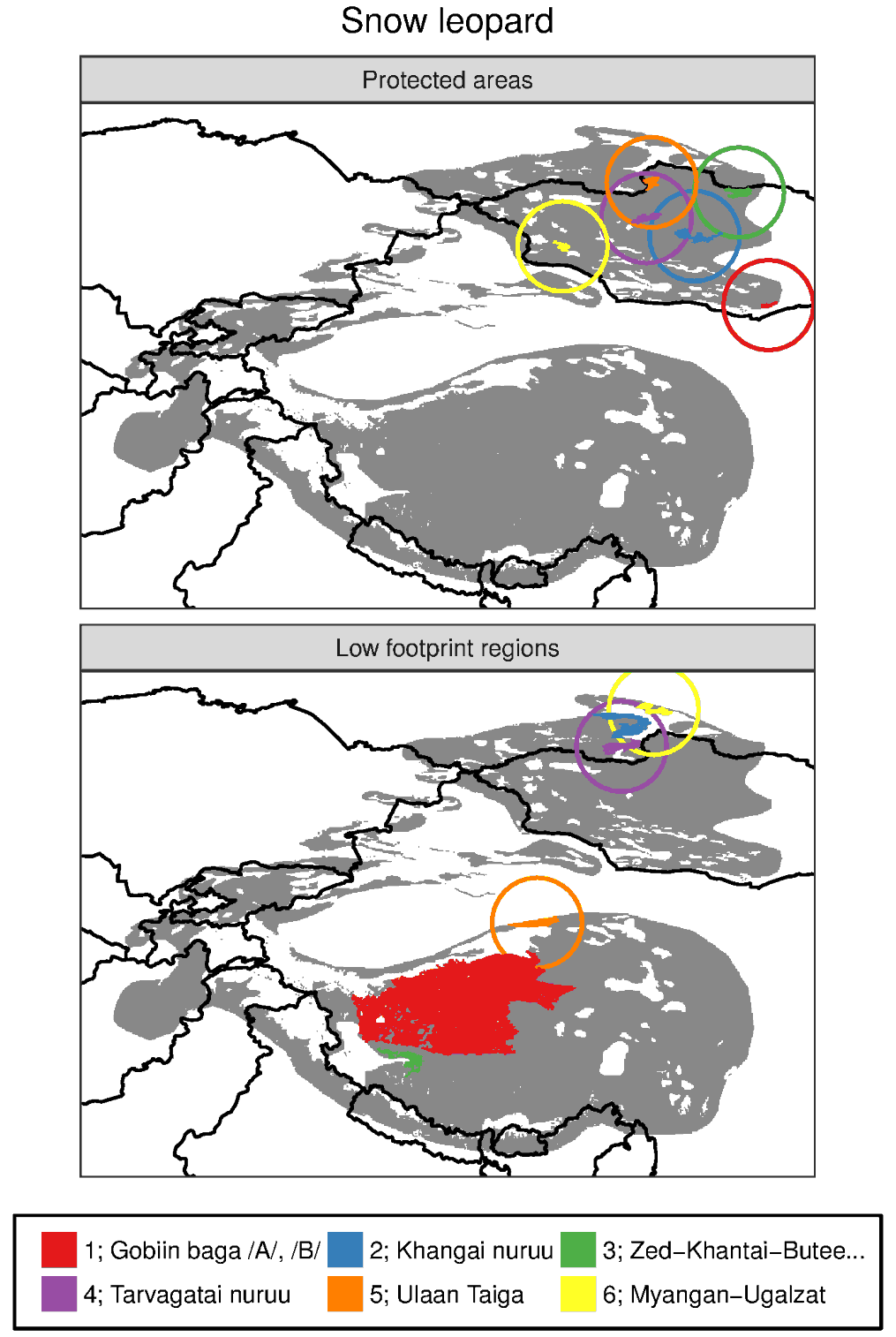

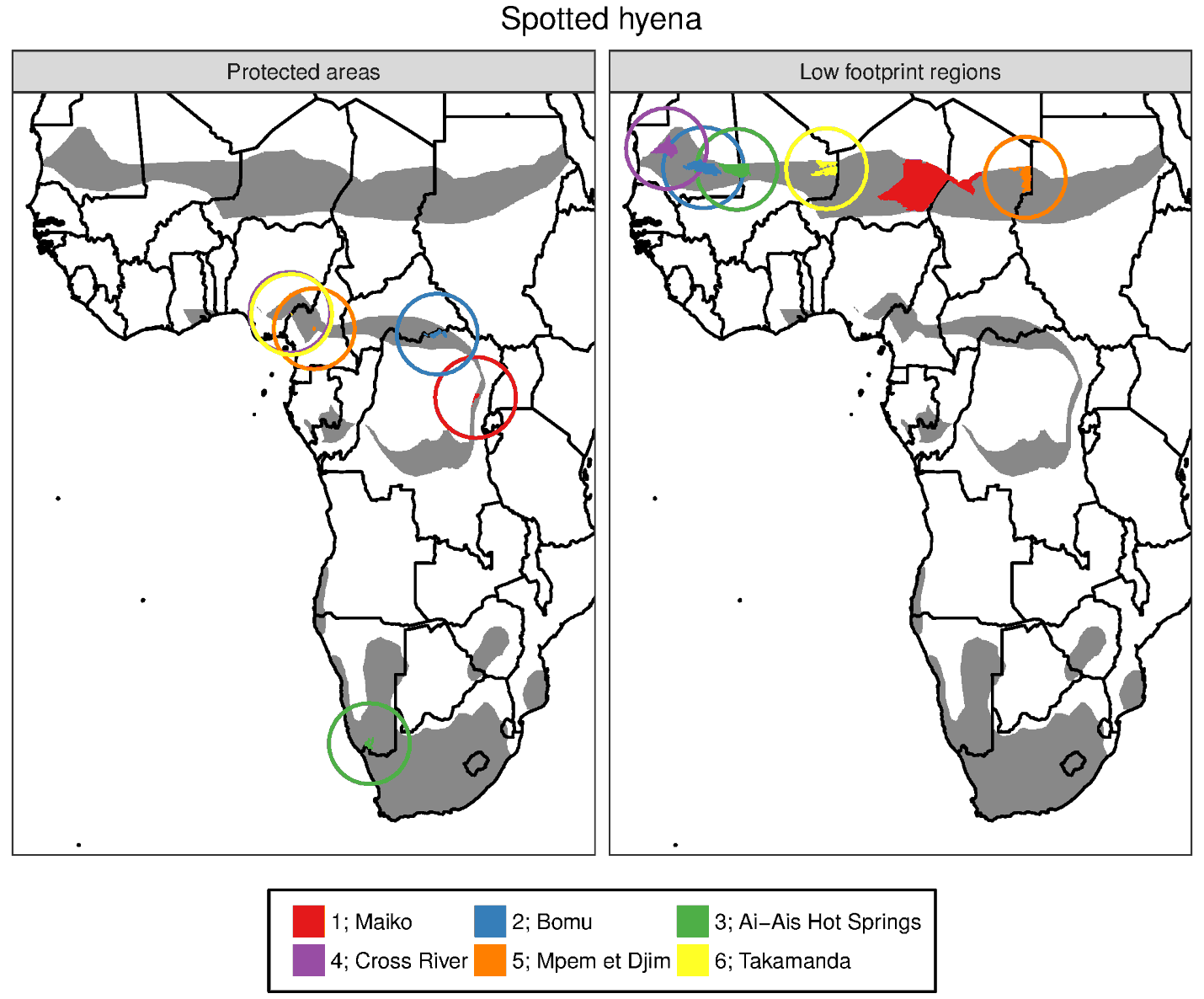

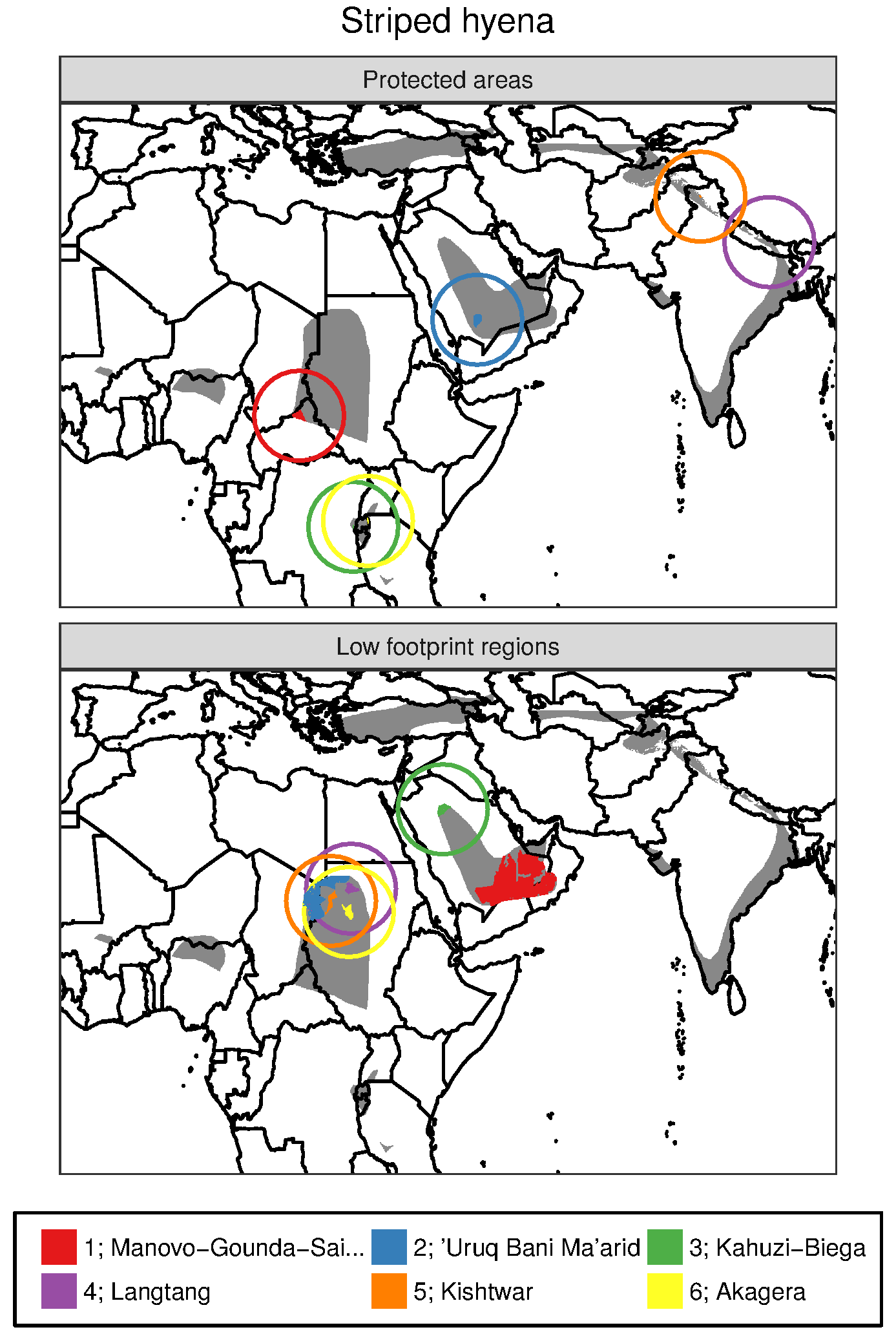

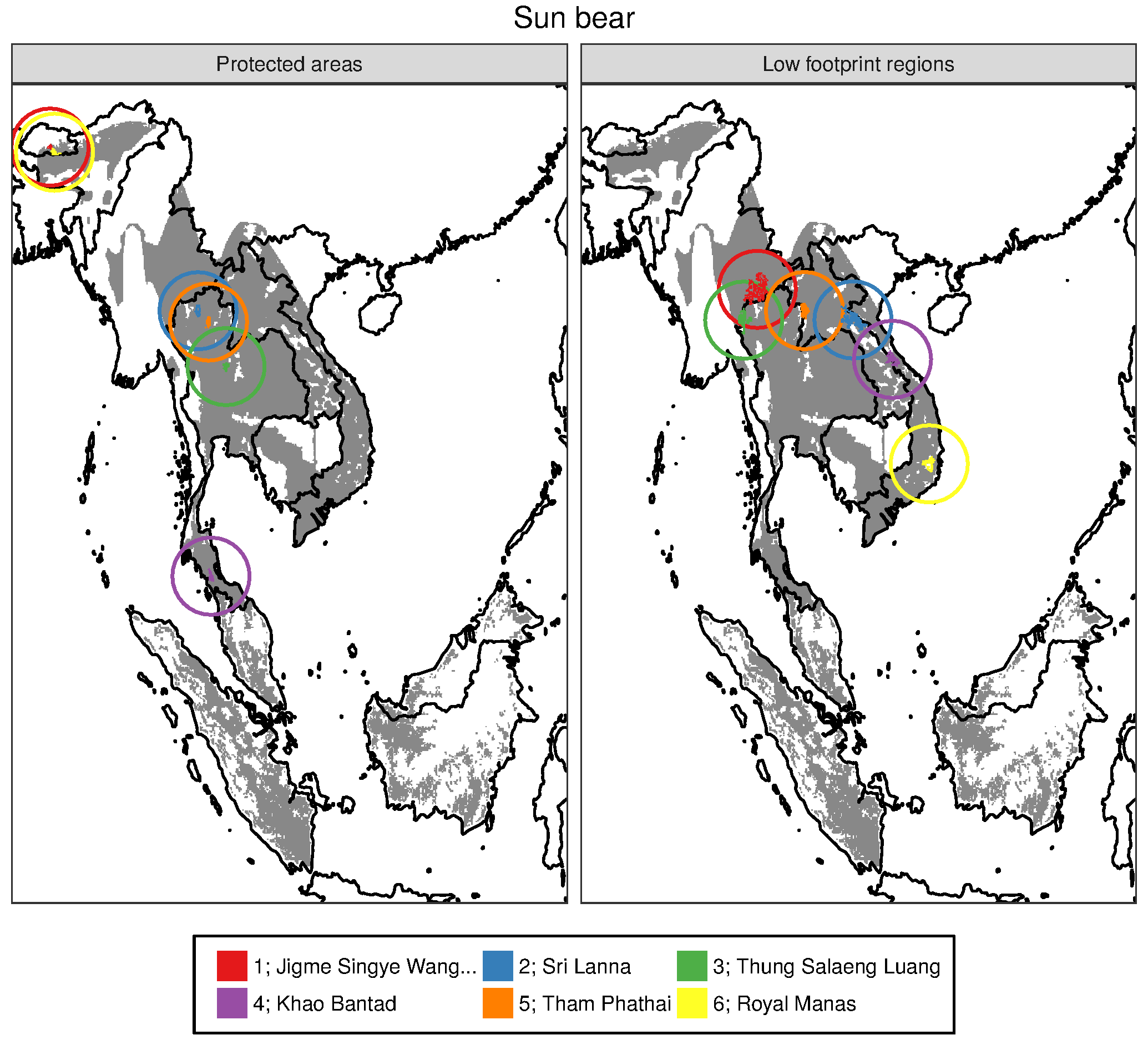

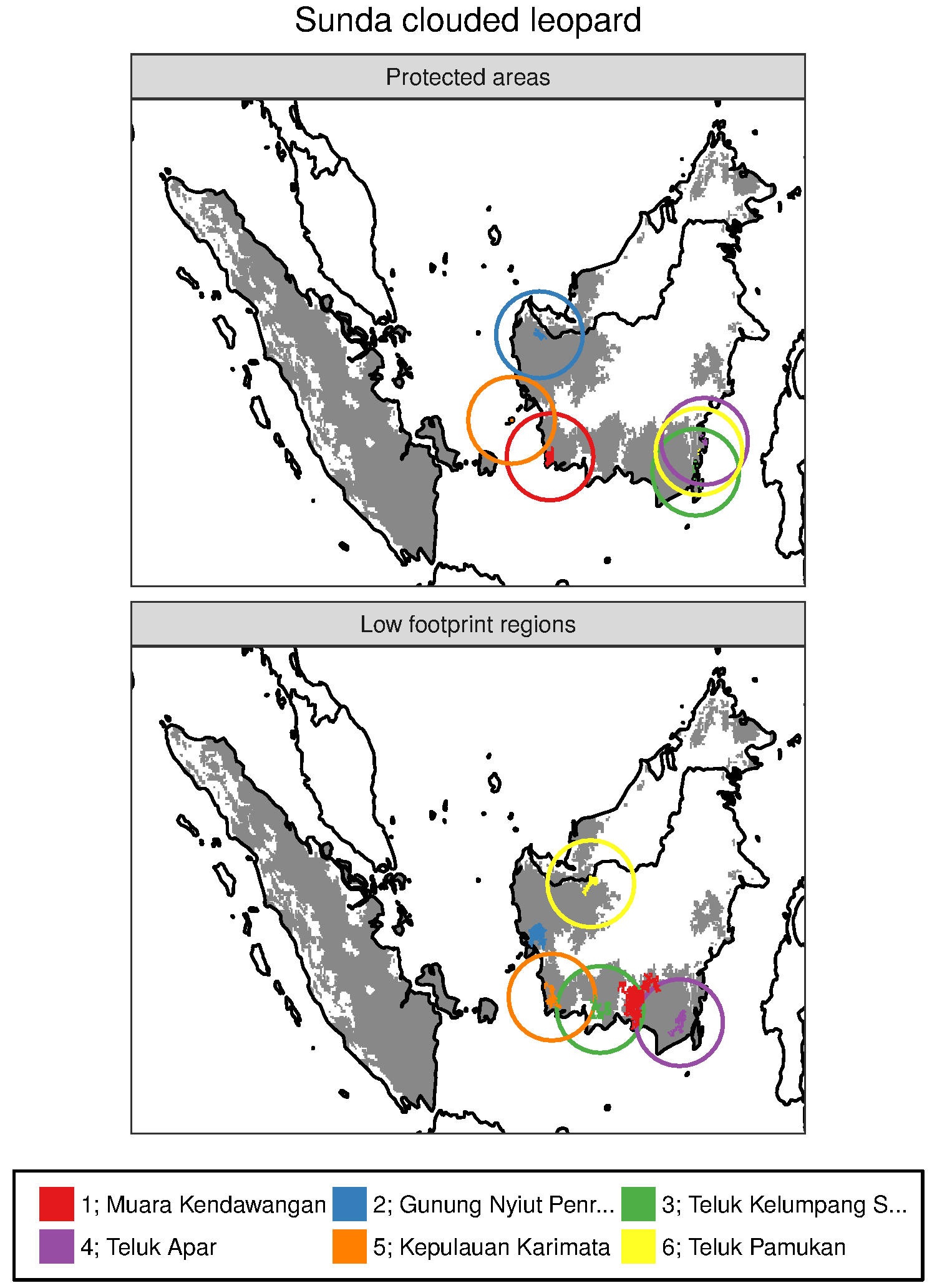

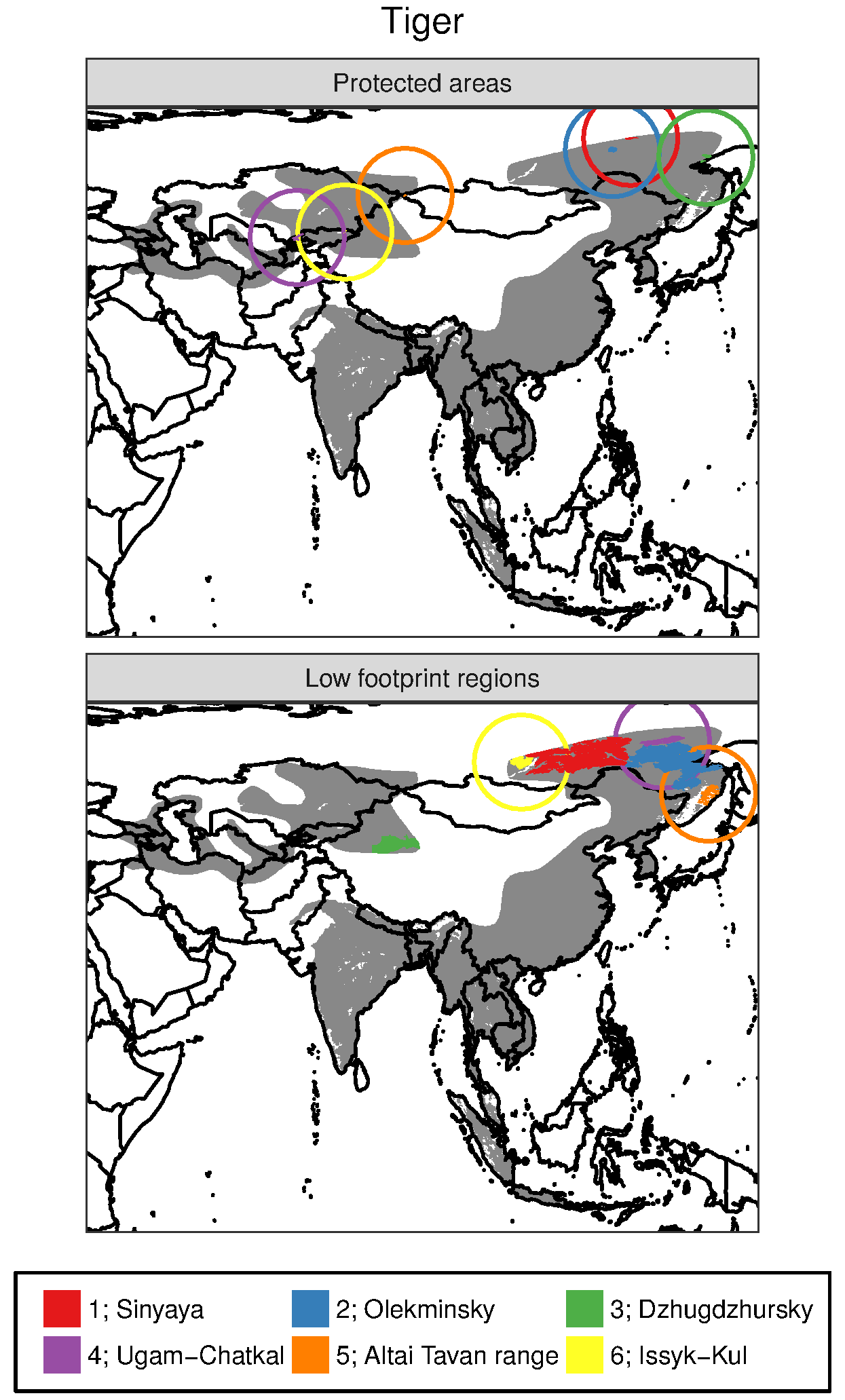
**

**
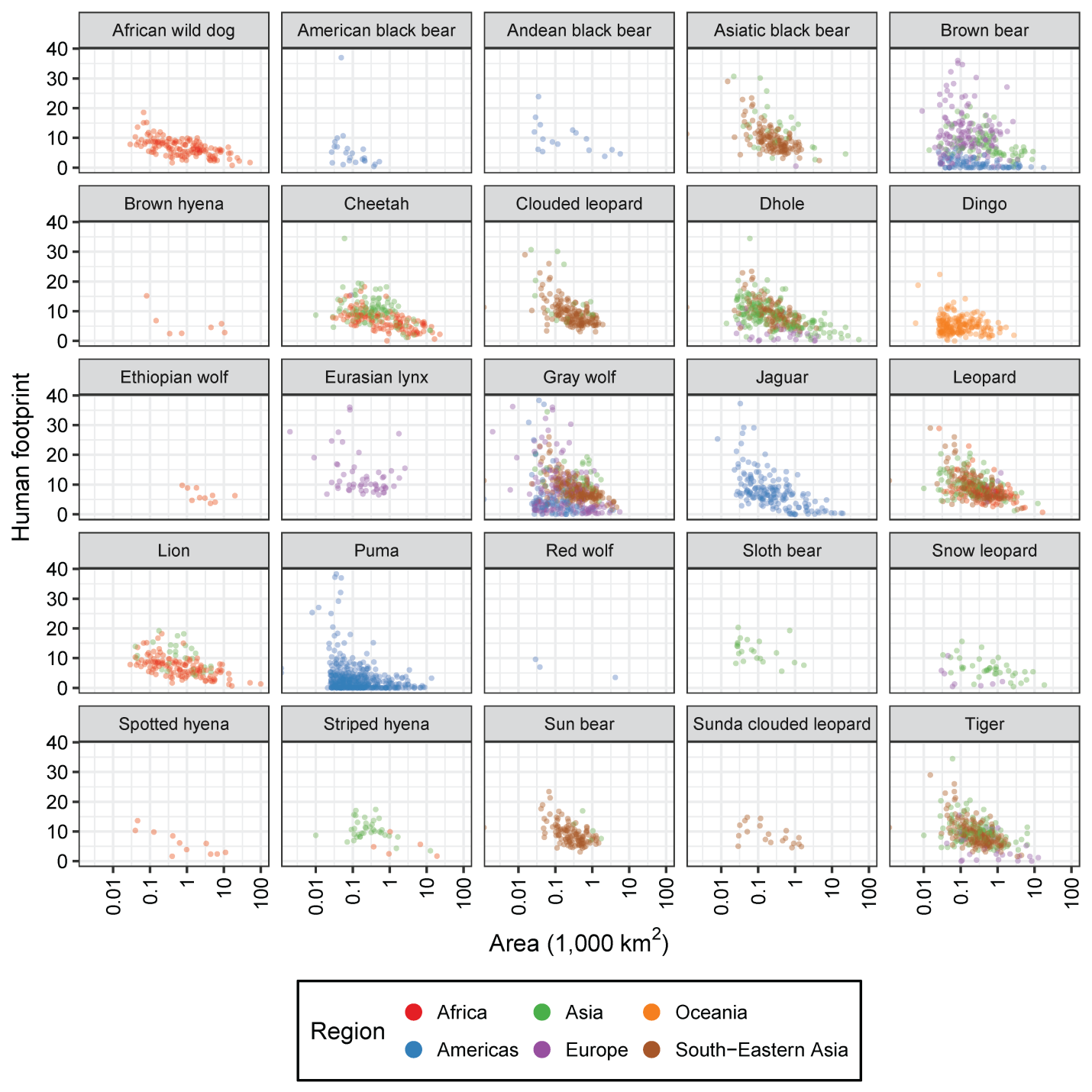
**

**Figure S3.** All strictly (category I-III) protected areas inside the ‘lost’ (historic minus current) ranges of each large carnivore species (without any manual validation). For each carnivore species, variables shown are the mean human footprint across the protected area and the region of the world. There are a total of 3,470 protected areas shown here. The species for which we identified the most protected areas were the gray wolf (n = 509), puma (n = 435), leopard (n = 353), dhole (n = 341), and tiger (n = 338), while the fewest protected areas were identified for the red wolf (n = 3), brown hyena (n = 7), Ethiopian wolf (n = 10), spotted hyena (n = 11), and Andean black bear (n = 16) (Figure S1). More than 100 protected areas were found in each of the world’s major geographic regions: Africa (n = 196), the Americas (n = 652), Asia (excluding South-Eastern Asia) (n = 3,41), Europe (n = 269), Oceania (n = 193), and South-Eastern Asia (n = 261).

**
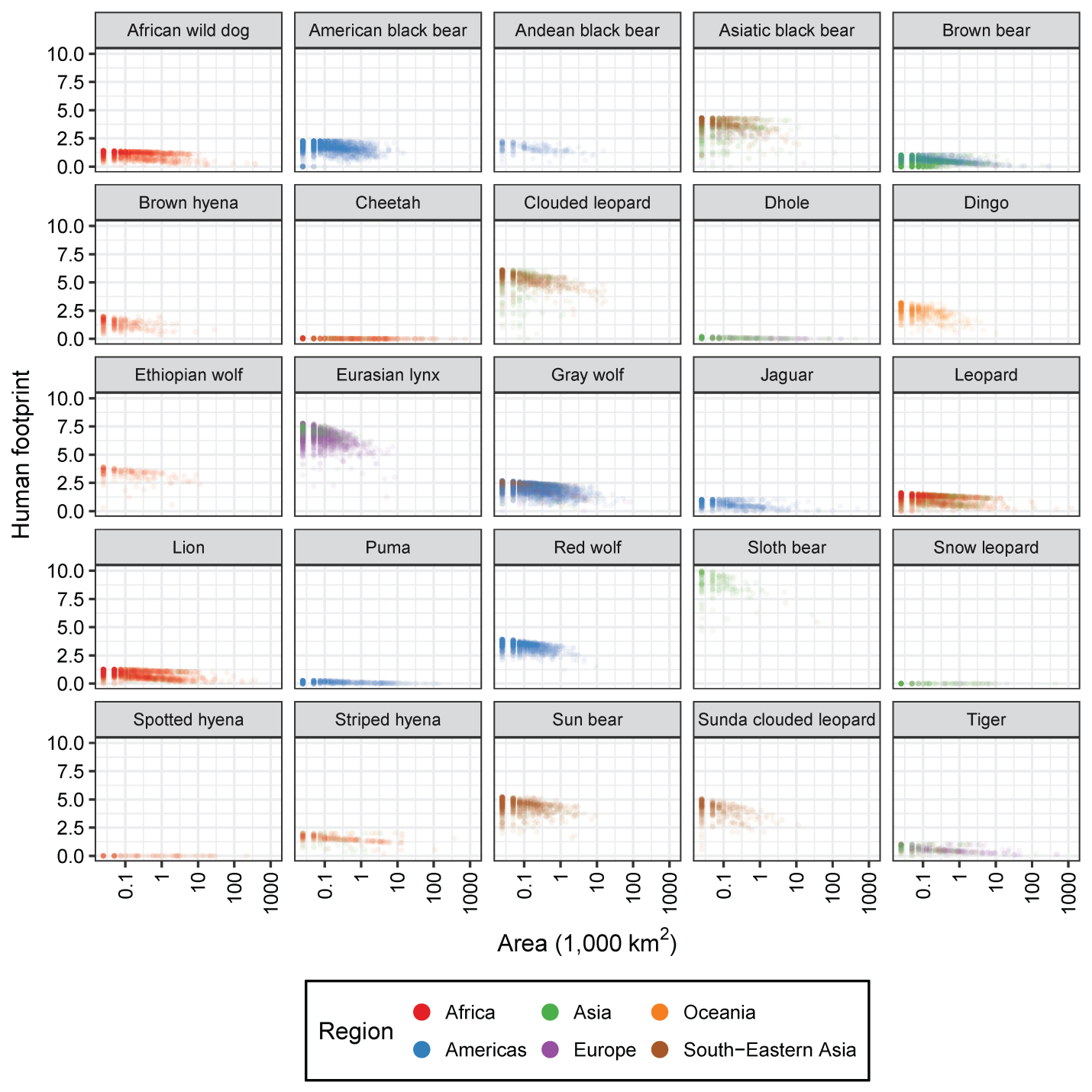
**

**Figure S4.** All contiguous low footprint regions inside the ‘lost’ (historic minus current) ranges of each large carnivore species. Low footprint regions were defined using the bottom 10% threshold for each species. For each carnivore species, variables shown are the mean human footprint across the protected area and the region of the world. A total of 22,234 low footprint regions were identified. The species for which we identified the most low footprint regions were the gray wolf (n = 3,544), brown bear (n = 2,366), American black bear (n = 1,987), Eurasian lynx (n = 1,884), and leopard (n = 1,825), while the fewest regions were identified for the spotted hyena (n = 96), snow leopard (n = 151), Andean black bear (n = 162), Ethiopian wolf (n = 165), and sloth bear (n = 184). Low footprint regions were found throughout the world: Africa (n = 4,833), the Americas (n = 8,507), Asia (excluding South-Eastern Asia) (n = 3,490), Europe (n = 2,725), Oceania (n = 476), and South-Eastern Asia (n = 2,203).
